# Electrochemiluminescence Drives Photodynamic Therapy In Vivo

**DOI:** 10.1002/advs.202512027

**Published:** 2025-12-22

**Authors:** Jia‐Bao Lin, Ling‐Ling Xu, Yong Liu, Hang Gao, Hong‐Yuan Chen, Jing‐Juan Xu

**Affiliations:** ^1^ State Key Laboratory of Analytical Chemistry for Life Science, School of Chemistry and Chemical Engineering Nanjing University Nanjing China; ^2^ School of Chemistry and Chemical Engineering Yangzhou University Yangzhou China

**Keywords:** electrochemiluminescence, in vivo, photodynamic therapy, tumor suppression

## Abstract

Electrochemiluminescence (ECL) holds significant promise in the biomedical field, but remains a challenge for in vivo photodynamic therapy (PDT). Herein, a wearable light source of ECL to drive PDT (ECL‐PDT) is developed, in which a flexible ECL device (ECLD) is fabricated using an ECL gel containing Ru(bpy)_3_Cl_2_ as the light‐emitting layer positioned between two transparent indium tin oxide‐coated substrates. By applying alternating current voltage, Ru(bpy)_3_^+^ and Ru(bpy)_3_^3+^ are alternately generated within the ECLDs, and then collide to produce excited species, followed by high‐efficiency and stable ECL emission. The ECLDs can further activate photosensitizers to produce reactive oxygen species through a resonance energy transfer process, ultimately leading to oxidative damage of cancer cells. As a result, such ECLDs demonstrate excellent antitumor efficacy on cells, and tumor growth is distinctly inhibited in a tumor‐bearing mouse model. This work not only provides a convenient and effective ECL‐PDT strategy for cancer treatment, but also expands the application of ECLD as a wearable photonic healthcare device.

## Graphical abstract

Electrochemiluminescence (ECL)‐driven photodynamic therapy (PDT) for in vivo cancer treatment is reported for the first time. The flexible ECL patch exhibits stable and intense luminance under alternating current voltage, enabling efficient photosensitization to generate reactive oxygen species that damage cancer cells, thus leading to significant suppression of tumor growth.

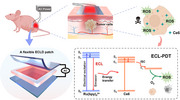

## 1 Introduction

Electrochemiluminescence (ECL), a light‐emitting process, is triggered by electrochemical reactions on the electrode surface, demonstrating high spatiotemporal controllability and operation [1, 2, 3, 4, 5, 6, 7, 8, 9]. As an output of optical signals, ECL has been applied in modern science, such as immunoassays [10, 11], biosensing [12, 13, 14, 15, 16], environmental monitoring [17, 18], single‐entity microscopy imaging [19, 20, 21, 22, 23, 24, 25], information encryption and decryption [26, 27], as well as optical displays [28, 29, 30]. Moreover, the development of phototherapeutic systems that enable personalized treatment for diseases is enhanced by ECL‐based light sources [31]. In 2018, Dai and coworkers first reported an ECL‐therapeutics system that utilized the ECL emitted from luminol to excite photosensitizers and generate reactive oxygen species (ROS) to kill pathogenic bacteria [32]. Later, ECL‐driven photodynamic therapy (PDT) for inducing cancer cell death was reported by our group, and the dynamic process of cellular apoptosis was monitored using ECL microscopy (ECLM) [33]. Despite promising in vitro results, liquid‐phase ECL confines its therapeutic utility. Therefore, there is an urgent need to develop solid‐state gel‐type ECL devices for personalized PDT.

Recently, an attempt has been made to apply wearable ECL devices (ECLDs) for antibacterial PDT. Zhang et al. developed a Ru(bpy)_3_^2+^‐based ECLD by integrating a conductive dual‐network hydrogel with flexible screen‐printed electrodes, which effectively inhibited bacterial infections and promoted the healing of chronic diabetic wounds [34]. The limited operational lifetime of the device, attributed to the irreversible consumption of coreactants, poses a significant barrier to its advancement in practical applications. Annihilation ECL of Ru(bpy)_3_^2+^ has brought promise for obtaining ECLDs with consistent and reliable signal output due to the theoretically recyclable nature of the annihilation reaction [35]. In 2014, Frisbie et al. reported that a polymeric light‐emitting gel‐based ECLD driven by alternating current (AC) within a simple sandwich architecture exhibited relatively stable luminescence [36]. Since then, a series of studies focusing on electronic skin [37], optical displays [38], and sensors [39] have been extensively explored. Especially, ECLDs fabricated on flexible substrates have captured attention for their skin conformability. For instance, Myoung et al. developed a wearable pressure‐sensitive light‐emitting sensor based on ECLD, enabling real‐time visualization of external stimuli [40]. To date, there have been rare reports on such solid‐state ECLDs for in vivo PDT.

**SCHEME 1:**
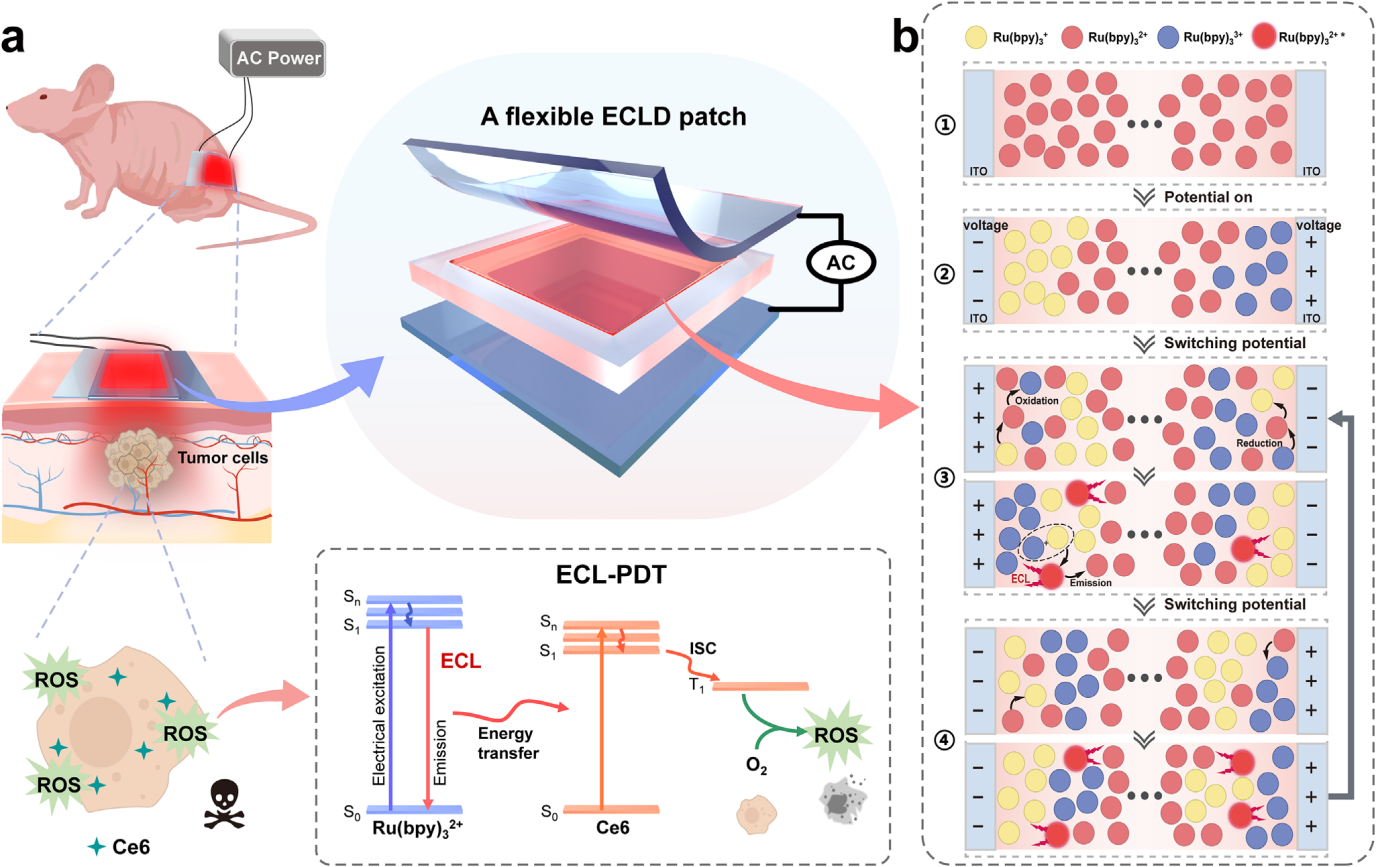
(a) Schematic illustration of the flexible ECLD patch for ECL‐PDT. (b) Schematic description of chemical species and their reactions near the electrode under AC voltage: ① no voltage applied; ② oxidation and reduction of Ru(bpy)_3_^2+^ upon the applied voltage; ③ the distribution of radical ions and ECL emission induced by a switch of voltage; ④ the re‐distribution of radical ions and light emission by another switching voltage.

In this work, by utilizing Ru(bpy)_3_^2+^‐based ECL gel as a light source, we fabricated a flexible ECLD patch for driving PDT in vivo (Scheme 1), and this ECLD exhibits high luminance and relatively stable operation. After establishing a tumor‐bearing mouse model of breast cancer, ECLD patch is stuck on the skin of the tumor position. Under an AC voltage condition, ECL gel emits light through the radiative transition of excited species Ru(bpy)_3_^2+*^ generated from the annihilation reactions between Ru(bpy)_3_^+^ and Ru(bpy)_3_^3+^ (Scheme 1b). Moreover, chlorin e6 (Ce6) is selected as a photosensitizer due to its high triplet yield and efficient energy transfer to oxygen, generating robust ROS under irradiation of the ECLD, which subsequently damages tumor cells. Significantly, compared with the control groups, the treatment group exhibits distinct antitumor effects in both in vitro and in vivo experiments. This flexible ECLD offers manipulable and portable approaches for PDT and indicates potential clinical applications for cancer treatment.

## 2 Results and Discussion

### 2.1 Preparation and Luminescent Properties of ECLD

**FIGURE 1:**
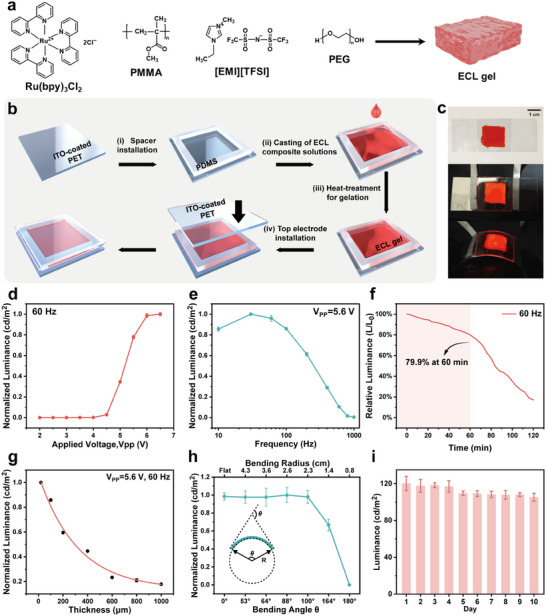
(a) Illustration of preparation of ECL gel composed of Ru(bpy)_3_Cl_2_, PMMA, [EMI][TFSI], and PEG. (b) Schematic of the fabrication process of the flexible ECLDs. (c) Photographs of fabricated ECLD and its light emission in the initial and bending states. (d) Normalized luminance of the ECLD as a function of the applied voltage (V_PP_) at 60 Hz (*n* = 3; mean ± SD). (e) Normalized luminance of the ECLD as a function of AC input frequency at V_PP_ = 5.6 V (*n* = 3; mean ± SD). (f) Relative luminance (L/L_0_, where L_0_ represents the initial luminance) of the ECLD during continuous operation for 120 min. (g) Normalized luminance of the ECLD as a function of thickness of ECL gel (*n* = 3; mean ± SD). (h) Normalized luminance of the ECLD as a function of the bending angle and radius (*n* = 3; mean ± SD). (i) ECL luminance of an ECLD emitting continuously for 5 min per day over a 10‐day period (*n* = 5; mean ± SD). The data in Figures f to i were collected at V_PP_ = 5.6 V and a frequency of 60 Hz.

ECL gel was prepared according to previous reports with minor revisions [36, 38], shown in Figure 1a. Ru(bpy)_3_Cl_2_, 1‐ethyl‐3‐methylimidazolium bis(trifluoromethylsulfonyl)imide ([EMI][TFSI]), poly(methyl methacrylate) (PMMA), and poly(ethylene glycol) (PEG) were co‐dissolved in dichloromethane (DCM) to obtain the composite solution. Ru(bpy)_3_Cl_2_ is utilized as the ECL luminophore. Ionic liquids [EMI][TFSI] are selected as supporting electrolytes owing to their higph ionic conductivity, while polymer PMMA regulates viscosity to promote gelation. Additionally, low‐molecular‐weight PEG can improve the ion transport of electrolytes and serve as a plasticizer to increase the elongation at break [41, 42, 43]. Next, the flexible ECLD was fabricated under ambient conditions illustrated in Figure 1b. The patterned polydimethylsiloxane (PDMS) was adhered to the bendable indium tin oxide (ITO)‐coated poly(ethylene terephthalate) (PET) substrates as a template for ECL gel. Subsequently, the prepared composite solution was cast onto the patterned substrate, followed by heat treatment to volatilize the DCM completely for gelation.

Notably, scanning electron microscopy (SEM) images indicate that the surface of the ECL gel is uniform without crystallization or precipitation (Figure S1). In addition, the viscosity of the ECL gel is measured to be 46.51 ± 0.68 Pa·s (Figure S2), signifying its soft‐solid nature [44]. And, the density of the ECL gel is calculated to be 1.64 g cm^−3^. Furthermore, another ITO‐coated PET electrode was placed on the ECL gel, and the ECLD with a simple structure was obtained. Figure 1c presents the fabricated ECLD under daylight without electrical operation (top), along with its light‐emitting states before (middle) and after (bottom) bending under electrical operation.

Next, we investigated the light‐emitting properties of our prepared flexible ECLD. Figure S3 illustrates the ECL characteristics of the ECLD under a square‐wave voltage signal with a peak‐to‐peak voltage (V_PP_) of 5.6 V and a frequency of 10 Hz. Impressively, strong ECL emission is observed for the ECLD, compared to 0.1 mM Ru(bpy)_3_^2+^/tri‐*n*‐propylamine (25 mM) system (Figure S4). It is well known that the ECL generation of ECLDs is dependent on the collision between the oxidizing and reductive species of Ru(bpy)_3_^2+^ near the same electrode surface. To achieve the optimal performance of ECLD, we investigated the effects of the applied AC voltage and frequency on luminance of the ECLDs. At a given frequency of 60 Hz, the ECLDs initiate light emission at V_PP_ around 4.1 V (Figure 1d), and the luminance increases progressively as the V_PP_ rises from 4.1 to 6.5 V. Notably, electrochemical decomposition of luminophores or the side reactions of electrolyte may occur at high V_PP_ [35, 45, 46]. To avoid rapid deterioration of ITO electrodes, a slightly lower V_PP_ of 5.6 V was adopted. Figure 1e shows the dependence of luminance on the applied frequency, indicating the optimal frequency range of 30–60 Hz. At a frequency of 60 Hz, the ECLD maintains 79.9% of its initial luminance after continuous operation for 60 min (Figure 1f), after which the luminance drops sharply. This is mainly ascribed to the progressive degradation of the ITO electrode during multiple reduction cycles, leading to decreased transparency and electrical conductivity, as well as the degradation of the electrolyte and luminophore after multiple redox cycles [35, 46, 47, 48]. Conversely, as for operation at a frequency of 30 Hz for 60 min, the luminance of the ECLD decreases rapidly with only 1.54% of the initial value remaining (Figure S5), which may be due to the short lifetime of redox species leading to an imbalance in the diffusion [49]. These results indicate that at a frequency of 60 Hz and V_PP_ of 5.6 V, the ECLD maintains a relatively high luminance during the initial 60 min of continuous operation, satisfying the requirement for 30 min of sustained illumination in subsequent treatments. Therefore, an applied voltage of 5.6 V and a frequency of 60 Hz were selected for the subsequent experiments (Video S1).

Subsequently, the concentrations of Ru(bpy)_3_^2+^ and PEG in the ECL gel were optimized (Figures S6 and S7). ECLD luminance increases with Ru(bpy)_3_Cl_2_ and PEG content and saturates at 9 wt.% and 5 wt.%, respectively. In addition, we observed that compared with PEG‐containing ECLD that retains a relatively stable luminance (Figure 1f), the PEG‐free ECLD manifests a pronounced luminance decay, falling to ∼10% of its initial value after 30 min of continuous operation (Figure S8). This might be due to the fact that low‐molecular‐weight PEG enhances ion transport and accelerates electrode/electrolyte kinetics, thereby improving luminous efficiency and long‐term stability [41, 42, 43].

In addition, it is observed that the luminance of the ECLD decreases with increasing thickness of the ECL gel (Figure 1g), which might be attributed to the lower volumetric resistivity of a thinner ECL gel. Under the same applied voltage, a thinner ECL gel results in a larger electric field at the electrode [37]. Thus, the ECLD with a thinner ECL gel can initiate luminescence at a lower applied voltage (Figure S9). To ensure that the light‐emitting area effectively covers the dimensions of the tumor, a thin and flexible ECLD patch was fabricated using an ECL gel with an area of 1.5 × 1.5 cm^2^ and a thickness of 20 µm. Under such conditions, the luminance of the ECLD can reach 125.5 cd/m^2^, which is higher than the electroluminescent device utilized in PDT with a luminance of 30.62 cd/m^2^ [50]; the power density is approximately 0.10 W/cm^2^, superior to or comparable to the previously reported value for inducing PDT [51, 52]. This implies that the ECLD we fabricated could effectively activate photosensitizer to generate ROS for damaging tumor cells. Then, the luminance of the ECLD under bending conditions was measured to evaluate the mechanical properties of the flexible ECLD patch as a wearable device (Figure 1h; Figure S10). Figure 1h depicts that the luminance of the ECLD remains stable at different bending angles (θ = 0°, 53°, 64°, 88°, and 100°), enabling the device to effectively conform to most of the curved surfaces of human skin. The luminance decreases rapidly upon further increasing the bending angle, attributed to the cracking of the ITO electrodes and the delamination of the ECL gel from the electrodes. In addition, four ECLDs we fabricated show no significant difference of ECL luminance (Figure S11), indicative of good reproducibility of these ECLDs. Furthermore, the ECLD exhibits satisfactory stability in luminescence intensity after operating for 5 min each day over a 10‐day monitoring period, with a high signal retainability of 87.6% compared to its initial signal (Figure 1i). This likely reflects diffusion‐driven replenishment of the luminophore in the ECL gel toward the electrode during the rest intervals of intermittent operation [29, 53, 54]. Overall, the high‐efficiency and stable light‐emitting properties of the wearable ECLD are obtained, which lays a foundation for therapeutic applications.

### 2.2 ROS Generation Evaluation of ECL‐PDT

**FIGURE 2:**
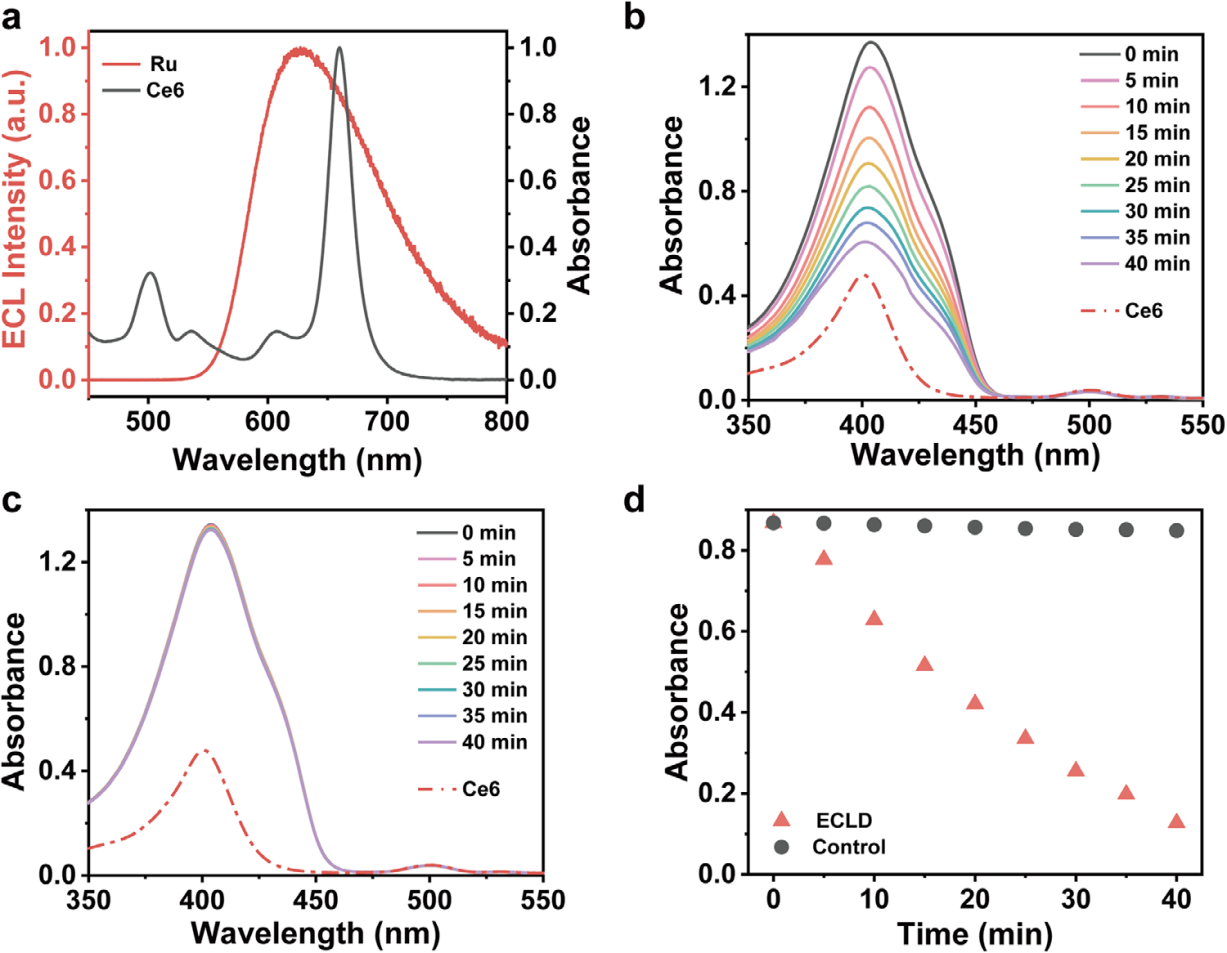
(a) The ECL emission spectrum of ECLD and absorption spectrum of Ce6. Absorption spectra of DPBF (50 µM) in the presence of Ce6 (10 µM) in H_2_O/ethanol (1:1, v/v), recorded every 5 min over a 40 min interval, with ECLD irradiation (b) or not (c); The red dashed line presents the absorption spectrum of Ce6 (10 µM) in H_2_O/ethanol (1:1, v/v). (d) The absorbance of DPBF at 400 nm under ECLD irradiation (red triangles) and non‐irradiation (black dots).

To verify the feasibility of PDT driven by our developed ECLD, we collected the ECL spectrum of Ru(bpy)_3_Cl_2_ and absorption spectrum of photosensitizer Ce6, and found a good overlap between them (Figure 2a). This indicates that the energy transfer can occur from Ru complexes to Ce6, and oxygen can be activated to form singlet oxygen (^1^O_2_) [55].

The generation of ^1^O_2_ under various irradiation time with ECLD was investigated, in which 1,3‐diphenylisobenzofuran (DPBF) is utilized as a detection probe. The experimental design is schematically shown in Figure S12. DPBF undergoes oxidation by ^1^O_2_ to form 1,2‐dibenzoylbenzene (DBB), resulting in a marked decrease in absorbance around 410 nm [56]. The absorption of the solution containing DPBF and Ce6 progressively decreases during continuous 40 min ECLD irradiation (Figure 2b), while the absorption spectra exhibit negligible changes in the absence of irradiation (Figure 2c). After subtracting the background absorbance of Ce6, it is easily observed that absorbance of the solution at 400 nm shows an 85% decrease after 40 min of ECLD irradiation (Figure 2d, red triangles), in contrast to the unirradiated control which shows almost unchanged signals (Figure 2d, black dots). Note that the mixture containing DPBF and Ce6 exhibits an absorbance maximum at 400 nm. These results suggest that the flexible ECLD as a wearable PDT patch can effectively activate the Ce6 to generate biocidal ROS.

### 2.3 In Vitro ECL‐PDT Effect

**FIGURE 3:**
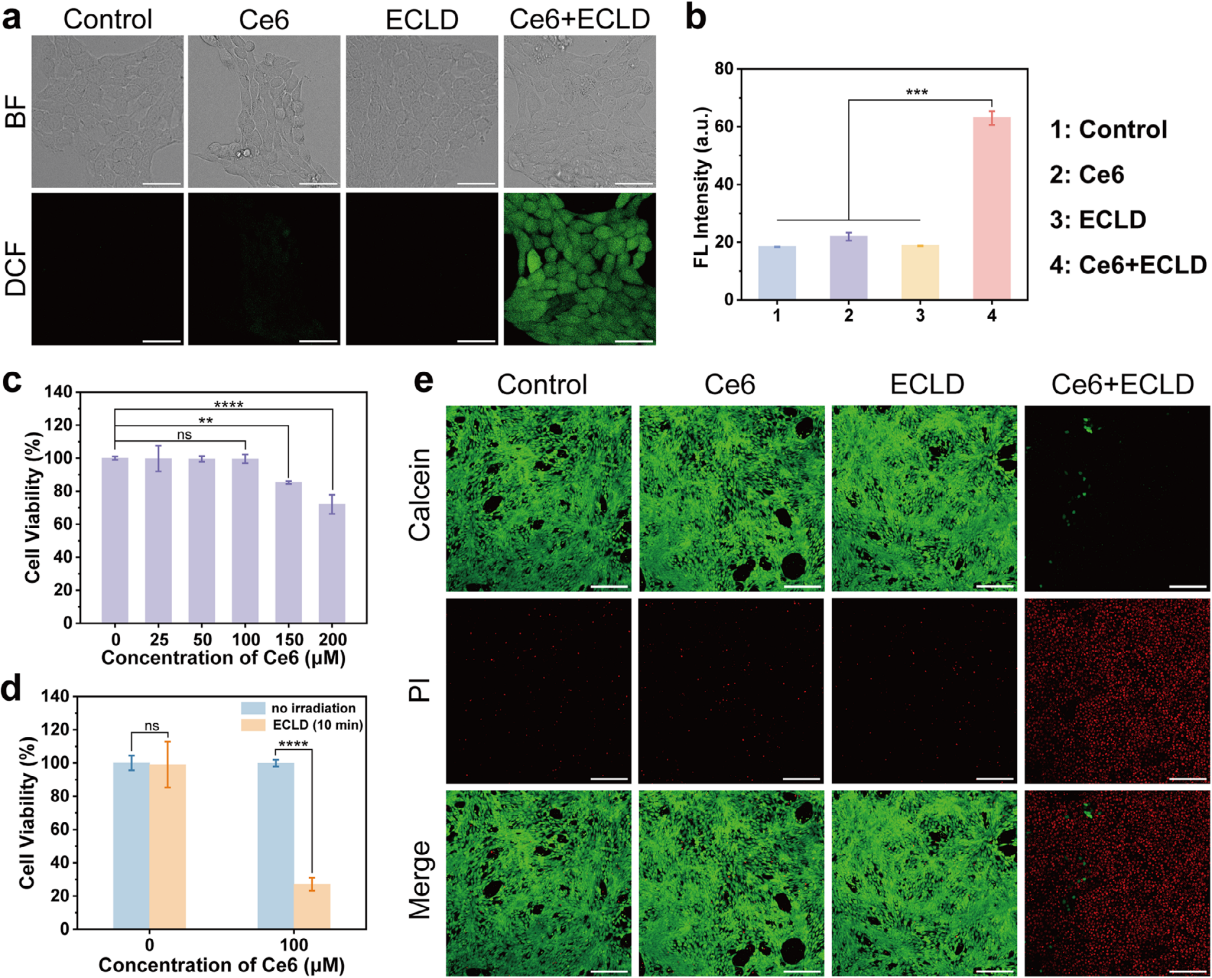
(a) Bright‐field (BF) and CLSM images of 4T1 cells stained with DCFH‐DA to detect ROS after different treatments. The scale bar is 50 µm. (b) The statistical analysis of the average DCF fluorescence intensity for the four groups (*n* = 3; mean ± SD). (c) Dark cytotoxicity of Ce6 toward 4T1 cells (*n* = 5; mean ± SD). (d) Cell viability of 4T1 cells incubated with either 0 or 100 µM Ce6 for 24 h, followed by ECLD irradiation for 10 min or no irradiation (*n* = 5; mean ± SD). (e) CLSM images of Calcein AM (green) and PI (red) co‐stained 4T1 cells after different treatments. The scale bar is 200 µm. Statistical significances were analyzed using one‐way ANOVA with Tukey's test. **p < 0.01, ***p < 0.001, ****p < 0.0001 and ns, no significant difference.

We further explored the effects of ECL‐PDT on tumor cells. The intracellular ROS generation in 4T1 cells was assessed by 2,7‐dichlorodihydrofluorescein diacetate (DCFH‐DA) that can be oxidized to form fluorescent intermediates. Briefly, 4T1 cells were incubated with 100 µM Ce6 for 8 h in the confocal culture dishes, and the cells could effectively take up Ce6 (Figure S13), followed by either 10 min of ECLD irradiation or no irradiation through the bottom of the dish (Figure S14). As shown in Figure 3a, the phosphate buffered saline (PBS), Ce6, and ECLD groups exhibit weak green fluorescence imaging of 4T1 cells, whereas distinct green fluorescence images are observed in the Ce6+ECLD group. Through statistical analysis of the average fluorescence intensity from confocal laser scanning microscopy (CLSM) images, it is easily found that the 2,7‐dichlorofluorescein (DCF) fluorescence intensity of the Ce6+ECLD group is highest (Figure 3b), indicating effective generation of ROS in cancer cells. Flow cytometry analyses further reveal that the Ce6+ECLD group can induce more apoptosis of tumor cells (Figure S15).

Additionally, the dark cytotoxicity of Ce6 was evaluated using the MTT assay with 4T1 cells (Figure 3c). At concentrations below 100 µM, Ce6 exhibits negligible dark cytotoxicity after incubation for 24 h. The viability of 4T1 cells remains above 80%, even when the concentration of Ce6 reaches up to 150 µM, suggestive of the low cytotoxicity of Ce6 under dark conditions. Furthermore, we also investigated the cell viability under ECLD irradiation. Compared to the no‐irradiation group, the viability of 4T1 cells treated with 100 µM Ce6 decreases significantly to 27.1% after only 10 min of ECLD irradiation (Figure 3d). These results show that ROS generated by ECLD irradiation is sufficient to effectively kill cancer cells. To intuitively distinguish the live and dead cells, calcein AM (green fluorescence) and PI (red fluorescence) were used to co‐stain 4T1 cells after various treatments (Figure 3e). As expected, almost all 4T1 cells died in the Ce6+ECLD group. In sharp contrast, only a few cell deaths are observed in the other three groups, i.e., the control, Ce6, and ECLD groups. These results demonstrate that ECL‐PDT exhibits significant cytotoxicity, indicating its potential applications in tumor treatment.

### 2.4 In Vivo Therapeutic Efficacy Evaluation of ECLD

**FIGURE 4:**
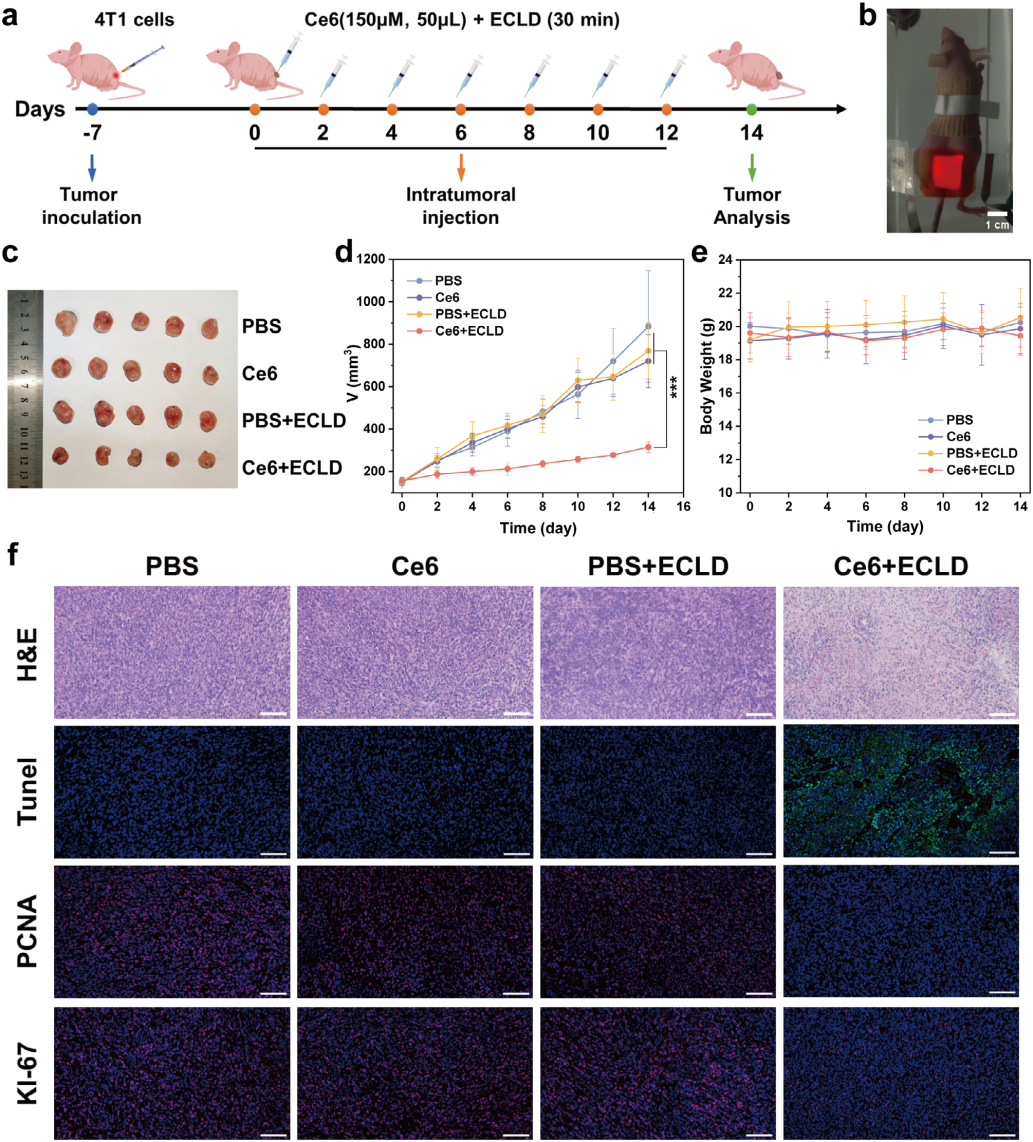
(a) Schematic of the in vivo treatment process of ECL‐PDT. (b) Photograph of the tumor‐bearing mouse treated with an ECLD patch. (c) Photograph of tumors in each group on day 14. The tumor growth (d) and body weight (e) curves of the mice after different treatments (*n* = 5; mean ± SD). (f) H&E, Tunnel, PCNA, and KI‐67 staining of the tumor tissues at 14th day of each treatment. The scale bar is 100 µm. Statistical significances were analyzed using one‐way ANOVA with Tukey's test. ***p < 0.001.

Given the encouraging antitumor efficacy of ECL‐driven photodynamic therapy at the cellular level, we further evaluated the in vivo efficacy of ECLD as a light source for PDT. The 4T1 tumor‐bearing BALB/c nude mice model was established by subcutaneous injection of 4T1 cells, and the treatment protocol is displayed in Figure 4a. Subsequently, 4T1 tumor‐bearing mice with a tumor volume of approximately 150 mm^3^ were randomly divided into four groups (*n* = 5 per group), i.e., PBS group, Ce6 group, PBS+ECLD group, and Ce6+ECLD group. During a 14‐day treatment period, each group of mice received treatments every two days. During each treatment session for the Ce6+ECLD group, Ce6 was injected into the tumor, followed by irradiation with an ECLD patch for 30 min (Figure S16). Figure 4b demonstrates the luminescence of ECLD patch on the tumor. The PBS group and the Ce6 group were respectively injected intratumorally with PBS or Ce6 solution in PBS, with no irradiation. For the PBS+ECLD group, the 4T1 tumor‐bearing mice were treated with the ECLD patch for 30 min after PBS injection. Throughout the treatment period, the tumor volume and body weight of mice in each group were recorded every two days. As illustrated in Figure 4c,d, the PBS, Ce6, and PBS+ECLD groups exhibit rapid tumor growth, whereas tumor growth in the Ce6+ECLD group is significantly suppressed, suggestive of the therapeutic effects in tumors. Notably, the average body weight of mice in each group remains stable over the 14‐day treatment period (Figure 4e), indicating that the treatment does not induce significant adverse effects.

To better evaluate the treatment efficacy, immunohistochemical analysis was performed. As can be seen from Figure 4f, hematoxylin and eosin (H&E) staining of tumor tissue sections demonstrates significant tumor cell damage in the Ce6+ECLD group, and the occurrence of apoptosis in the tumor cells is further validated by terminal deoxynucleotidyl transferase dUTP nick end labeling (TUNEL) staining. Additionally, proliferating cell nuclear antigen (PCNA) and KI‐67 staining results indicate a marked inhibition of tumor cell proliferation in the Ce6+ECLD group. The above results demonstrate that the ECLD‐driven PDT induces apoptosis in cancer cells, exhibiting a significant tumor‐suppressive effect. It is noted that H&E staining of major organs (heart, liver, spleen, lung, and kidney) reveals no evident histopathological damage (Figure S17), suggestive of the favorable biosafety profile of ECL‐PDT.

To assess the thermal effects during ECLD operation, infrared (IR) thermography was further recorded. As shown in Figure S18, the temperature of the ECLD patch remains essentially unchanged after 30 min of continuous ECLD irradiation, confirming that the ECLD exerts negligible heating. Consequently, the ECLD functions as a non‐thermal light source and affords reliable safety for prolonged applications.

## 3 Conclusions

In summary, we developed a novel ECL‐PDT strategy for cancer treatment in vivo, in which electricity‐driven flexible ECLDs were utilized as a light source. The ECLDs composed of Ru(bpy)_3_^2+^ complex are easily fabricated under ambient conditions, and exhibit efficient and stable ECL emission at low voltage with an adequate operational lifetime. Through activation of Ce6 by ECL emission of Ru(bpy)_3_^2+^, accompanied by the generation of ROS, the ECLDs we developed demonstrate efficient antitumor efficacy in both in vitro and in vivo. ROS‐triggered cell apoptosis is confirmed via immunohistochemical imaging. Impressively, the ECL‐PDT treatment of cancer possesses high biosafety and flexible modulation. This study offers new opportunities for exploring in vivo ECL‐based cancer therapeutics and paves the way for the application of ECLDs in the biomedical field. Structural design of flexible ECL devices, and research on their applications in biomedicine are currently underway in our laboratory.

## 4 Experimental Section

### 4.1 Materials

All commercially available chemicals and materials were used without any additional purification. Ru(bpy)_3_Cl_2_, PMMA with an average molecular weight of ∼350 k, and ITO‐coated PET (60 Ω sq^−1^) were obtained from Sigma–Aldrich Co. (Shanghai, China). [EMI][TFSI], PEG‐400, and DPBF were bought from TCI Development Co., Ltd (Shanghai, China). DCM was ordered from Sinopharm Chemical Reagent Co., Ltd. (Shanghai, China). The PDMS base and curing agent (Sylgard 184) were obtained from Dow Corning (Midland, MI, USA). mPEG2000‐Ce6 was provided by Xi'an Ruixi Biological Technology Co. Fetal bovine serum (FBS) was purchased from Gibco Life Technologies, while PBS, Roswell Park Memorial Institute (RPMI‐1640) medium, MTT Cell Proliferation and Cytotoxicity Assay Kit, and Hoechst 33 342 stain were purchased from KeGen Biotech Co., Ltd. (Nanjing, China). Reactive Oxygen Species Assay Kit, Matrix‐Gel Basement Membrane Matrix (High Concentration, without phenol red) and Calcein/PI Cell Viability/Cytotoxicity Assay Kit were ordered from Beyotime Biotechnology (Shanghai, China). Ultrapure water (≥18 MΩ cm^−1^) was produced using a Milli‐Q purification system (Millipore, MA). All chemicals employed in this study met analytical‐grade standards.

### 4.2 Apparatus and Characterization

Electrochemical and ECL measurements of the ECLD were obtained on a CHI 600e electrochemical workstation (CH Instruments Inc., China) in cooperation with a Home‐made HTR‐ECL analyzer. The applied AC voltage was supplied through an UTG1022X arbitrary waveform generator (UNI‐T., China). Luminance outputs of ECLD were recorded with a SM218E screen luminance meter (Sanpometer, China). The power density of the device was acquired on a UT385 laser power meter (UNI‐T., China). The UV–vis absorption spectrum was collected using a UV‐3600 UV–vis‐NIR spectrophotometer (Shimadzu, Kyoto, Japan). Microscopic morphology was examined by acquiring scanning electron microscopy (SEM) images with an S‐4800 scanning electron microscope (Hitachi, Tokyo, Japan). The viscosity experiment was carried out on a strain‐controlled ARES G2 rheometer (TA Instruments, New Castle, DE, USA). Confocal fluorescence images of cells were carried out using TCS SP8 confocal microscopes (Leica, Germany). Flow cytometric analysis was performed on a Coulter FC‐500 flow cytometer (Beckman‐Coulter). MTT cytotoxicity assays were recorded on a Thermo Scientific Varioskan Flash spectrophotometer (Thermo Fisher Scientific, U.S.A.). H&E tissue sections were imaged with an MP‐225 3D micromanipulator (Sutter Instrument, CA) combined with an Olympus/Nikon inverted fluorescence microscope (Ti2‐E, Japan). Fluorescence staining images of the tumor tissues were acquired on TCS SP8 confocal microscopes (Leica, Germany). The infrared thermal images were captured using HIKVISION (H16, Hangzhou Hikvision Digital Technology Co., Ltd.).

### 4.3 Synthesis of ECL Gel

The ECL gel was prepared on the patterned substrate through a solution‐casting process [36]. To obtain the ECL composite solutions, PMMA and [EMI][TFSI] (1:5 w/w) were dissolved in dichloromethane, followed by the incorporation of 8 wt.% Ru(bpy)_3_Cl_2_ and 5 wt.% PEG‐400 [38]. For forming the ECL gel, the ECL composite solutions were heated on a hot plate at 70°C for 50 min. Residual solvent was subsequently removed by placing the samples in a vacuum oven at 25°C.

### 4.4 Viscosity of ECL Gel

A 25 mm‐diameter parallel plate with a fixed gap of 0.5 mm was used in the linear viscoelastic measurements. The viscosity was measured across a shear rate range from 0.01 to 0.5 s^−1^, and small amplitude oscillatory shear from 100 to 0.1 rad s^−1^ was performed at a temperature of 23°C with 5% strain.

### 4.5 Fabrication of the ECLD

The ECL devices were fabricated in ambient air with the simple structure of ITO/ECL gel/ITO. First, the ITO‐coated PET was washed by sonication sequentially in acetone, isopropyl alcohol, ethanol, and ultrapure water for 5 min each, followed by a 15 min UV‐ozone surface treatment. Next, the patterned PDMS as a spacer as well as a template was stuck on the ITO‐coated PET substrates to define the emissive area (1.5 cm × 1.5 cm) and the thickness (∼20 µm) of the ECL gel. Then, the ECL composite solutions were cast on the patterned ITO‐coated substrate and allowed to gel by heating at 70 °C for 50 min. Finally, the second ITO‐coated PET was attached on the ECL gel to form the ECLD. To avoid any effects of moisture, ECL devices were kept in a vacuum desiccator before measurements and use.

### 4.6 ECL Spectrum

The ECL emission spectrum of ECLD was obtained by a self‐made ECL spectrum analyzer consisting of an UTG1022X arbitrary waveform generator and a FLS‐980 fluorescence spectrophotometer. The ECL spectrum of the ECLD was recorded under a square wave voltage signal (V_PP_ = 5.6 V) at a frequency of 60 Hz for 10 s.

### 4.7 Detection of Reactive Oxygen Species

1,3‐Diphenylisobenzofuran (DPBF) was selected as a probe to evaluate the time‐dependent production of ROS by Ce6 under the ECLD light treatment. Briefly, only Ce6 (10 µM) and DPBF (50 µM) were added to 3 mL of H_2_O/ethanol (1:1, v/v). Using ECLD irradiation (60 Hz, V_PP_ = 5.6 V), the absorption spectra of the solution were measured every 5 min by a UV–vis spectrophotometer over a time interval of 40 min. Then, the decrease in DPBF absorption (λ_max_ = 400 nm) was evaluated and plotted with time.

### 4.8 Cell Culture

Culture of 4T1 cells was performed in RPMI‐1640 supplemented with 10% FBS, streptomycin (100 µg mL^−1^), and penicillin (100 U mL^−1^). The cells were incubated at 37°C in a humidified 5% CO_2_ atmosphere.

### 4.9 MTT Cytotoxicity Assay

The cytotoxicity of Ce6 to 4T1 cells was assessed using MTT assay under dark conditions. 4T1 cells were seeded into 96‐well plates and allowed to adhere and proliferate overnight. Then, the culture medium was replaced with fresh medium containing various concentrations of Ce6 (0, 25, 50, 100, 150, and 200 µM). After a 24 h incubation period, the supernatant in each well was removed, and 50 µL MTT reagent was added, followed by a further 4 h incubation at 37 C. The MTT solution was carefully aspirated, and the formed formazan crystals in each well were completely dissolved by 150 µL DMSO. Absorbance at 490 nm was measured for each well using a Thermo Scientific Varioskan Flash spectrophotometer. In addition, the cell viability of 4T1 cells (24 h incubation with 0 or 100 µM Ce6) under ECLD irradiation (60 Hz, V_PP_ = 5.6 V, 0 or 5 min) was also determined using MTT assay.

### 4.10 Detection of Intracellular ROS

The probe DCFH‐DA was used to detect intracellular ROS. After entering the cells, DCFH‐DA was hydrolyzed by intracellular esterase to form DCFH, which is non‐fluorescent. In the presence of ROS, DCFH was subsequently oxidized to the green‐emitting fluorophore DCF, enabling the visualization of intracellular ROS. 4T1 cells were seeded in confocal culture dishes and cultured overnight. Then, 4T1 cells were incubated with either RPMI 1640 medium or Ce6 (100 µM) for 8 h, followed by a 20 min incubation with DCFH‐DA at 37 °C. After incubation, the cells were washed three times with PBS and irradiated with or without ECLD (60 Hz, V_PP_ = 5.6 V) for 10 min. Finally, the treated cells were immediately observed by a TCS SP8 confocal microscope with the excitation wavelength of 488 nm, and emission collection wavelength from 510 to 600 nm.

### 4.11 Flow Cytometry

After being seeded into 6‐well plates and incubated overnight, 4T1 cells were exposed to either RPMI 1640 medium or Ce6 (100 µM) for 8 h. DCFH‐DA staining was then carried out at 37 °C for 20 min. After triple PBS washing, the cells were irradiated with or without ECLD (60 Hz, V_PP_ = 5.6 V) for 10 min. Following their light irradiation, cells were washed again with PBS and detached using trypsin for 3 min at 37 °C/5% CO_2_. The cell pellets were then collected and resuspended in 500 µL of PBS. The fluorescence signal of intracellular DCF was measured on a Coulter FC‐500 flow cytometer (Beckman‐Coulter), and Flow Jo software was used for data analysis.

### 4.12 Live/Dead Cell Staining

Live/dead cell staining was performed using a Calcein/PI cell viability and cytotoxicity assay kit. 4T1 cells cultured in the confocal culture dishes for 24 h were divided into 4 groups (Control, Ce6, ECLD, and Ce6+ECLD). Each group of 4T1 cells was incubated with RPMI 1640 medium or 100 µM Ce6 for 24 h, followed by exposure to ECLD irradiation (60 Hz, V_PP_ = 5.6 V, 10 min) or no irradiation. After 1 h, 4T1 cells were washed twice with PBS and subsequently incubated with Calcein AM and PI for 30 min at 37 °C. Following triple PBS washing, the live/dead cell staining fluorescence imaging was captured using a TCS SP8 confocal microscope. Calcein‐AM produced green fluorescence (E_x_/E_m_ = 494/517 nm), whereas PI emitted red fluorescence (E_x_/E_m_ = 535/617 nm).

### 4.13 Animal Model

Female BALB/c nude mice (5–6 weeks old, ∼19 g body weight) were provided by Laboratory Animal Center (Nanjing Xuanwu District HFK Biological Products Center). All animal studies complied with the institutional guidelines and were reviewed and approved by the Animal Care and Use Committee of the Southeast University Laboratory Animal Center (No: 20 240 306 010). Tumor‐bearing models were established by subcutaneously inoculating 2 × 10^6^ 4T1 cells, suspended in 100 µL of a 1:1 PBS/Matrix‐Gel solution, into the right thigh of each nude mouse.

### 4.14 In Vivo Tumor Inhibition by ECL‐PDT

Upon reaching a tumor volume of about 150 mm^3^, the mice were randomly assigned to four groups (*n* = 5 for each group): 1) PBS (intratumoral injection, 50 µL); 2) Ce6 (intratumoral injection, 50 µL, 150 µM); 3) PBS (intratumoral injection, 50 µL) + ECLD (60 Hz, V_PP_ = 5.6 V, 30 min); 4) Ce6 (intratumoral injection, 50 µL, 150 µM) +ECLD (60 Hz, V_PP_ = 5.6 V, 30 min). Typical treatments were performed every two days, totally for 14 days. During 14 days, the tumor dimensions (length and width) and body weight were measured every other day. Tumor volume (V) was calculated as the following formula:

(1) $$V_{x}=\frac{L_{x}\times W_{x}^{2}}{2}$$

Here, L, W represent the tumor length and width measured after x day treatment. The mice were sacrificed after 14‐day post‐treatment, and their tumors were removed, photographed, and collected for immunohistochemical analysis. The major organs, including the heart, liver, spleen, lungs, and kidneys, were harvested and stored in 4% paraformaldehyde solutions.

### 4.15 Tissue Staining

After fixation with 4% paraformaldehyde solutions overnight, both the tumors and the major organs were paraffin‐embedded and subsequently sectioned. Tumor sections were processed for H&E (hematoxylin and eosin), Tunnel (terminal deoxynucleotidyl transferase dUTP nick end labeling), KI‐67 (proliferative marker), and PCNA (proliferating cell nuclear antigen) staining. For biosafety assessment, H&E staining was also performed on sections of the major organs.

### 4.16 Statistical Analysis

Data were shown as mean ± standard deviation (SD) from ≥ 3 replicates. Statistical analyses were performed using one‐way analysis of variance (ANOVA) with Tukey's test. Significance was defined as *p* < 0.05, with increasing levels of significance indicated as *p < 0.05, **p < 0.01, ***p < 0.001, and ****p < 0.0001. Origin and Excel software were used for statistical processing. Microscopy images were analyzed by ImageJ Fiji. FlowJo software was used for flow cytometric analysis.

## Conflicts of Interest

The authors declare no conflicts of interest.

## Supporting information




**Supporting File 1**: advs73531‐sup‐0001‐SuppMat.pdf.

**Supporting File 2**: advs73531‐sup‐0002‐VideoS1.mp4.

## Data Availability

The data that support the findings of this study are available in the supplementary material of this article.

## References

[1] Z. Ding , B. M. Quinn , S. K. Haram , L. E. Pell , B. A. Korgel , and A. J. Bard , “Electrochemistry and Electrogenerated Chemiluminescence from Silicon Nanocrystal Quantum Dots,” Science 296 (2002): 1293–1297. 12016309 10.1126/science.1069336

[2] H. Liu , A. Hussain , Y. T. Zholudov , D. V. Snizhko , N. Sojic , and G. Xu , “Self‐Powered Electrochemiluminescence for Imaging the Corrosion of Protective Coating of Metal and Quantitative Analysis,” Angewandte Chemie, International Edition 63 (2024): 202411764. 10.1002/anie.202411764 39048514

[3] N. S. Adamson , S. J. Blom , E. H. Doeven , et al., “Electrochemiluminescence Enhanced by a Non‐Emissive Dual Redox Mediator,” Angewandte Chemie, International Edition 63 (2024): 202412097. 10.1002/anie.202412097 39136339

[4] Y. Wang , S. Zhou , Y. Zheng , et al., “Measurements of Local pH Gradients for Electrocatalysts in the Oxygen Evolution Reaction by Electrochemiluminescence,” Journal of the American Chemical Society 147 (2025): 19380–19390. 40388601 10.1021/jacs.5c04896

[5] Y. Xu , X. Huang , Y. Wang , et al., “Controllable and Low‐Loss Electrochemiluminescence Waveguide Supported by a Micropipette Electrode,” Journal of the American Chemical Society 146 (2024): 5423–5432. 38354221 10.1021/jacs.3c12913

[6] Z.‐C. Shen , Y.‐F. Chen , Y.‐Q. Chai , J.‐L. Liu , and R. Yuan , “Entire Near‐Infrared‐I Electrochemiluminescence Enhancement of Gold Nanoclusters,” Angewandte Chemie, International Edition (2025): 202509884. 10.1002/anie.202509884 40468169

[7] X. Wei , K. Chu , J. R. Adsette , et al., “Nanocluster Transformation Induced by SbF6– Anions toward Boosting Photochemical Activities,” Journal of the American Chemical Society 144 (2022): 20421–20433. 36260434 10.1021/jacs.2c08632

[8] H. Gao , S. Y. Shi , S. M. Wang , et al., “Aggregation‐Induced Delayed Electrochemiluminescence of Organic Dots in Aqueous Media,” Aggregate 5 (2024): 394.

[9] Y. Wang , T. Liu , S. Yu , et al., “Polychromatic Electrochemiluminescence Imaging of Single Heteroligand Metal–Organic Crystals,” Angewandte Chemie, International Edition 64 (2025): 202501151. 10.1002/anie.202501151 40033945

[10] X. Yang , J. Hang , W. Qu , et al., “Gold Microbeads Enabled Proximity Electrochemiluminescence for Highly Sensitive and Size‐Encoded Multiplex Immunoassays,” Journal of the American Chemical Society 145 (2023): 16026–16036. 37458419 10.1021/jacs.3c04250

[11] A. Zanut , F. Palomba , M. Rossi Scota , et al., “Dye‐Doped Silica Nanoparticles for Enhanced ECL‐Based Immunoassay Analytical Performance,” Angewandte Chemie, International Edition 59 (2020): 21858–21863. 33000888 10.1002/anie.202009544

[12] W. Fu , X. Wang , X. Ying , et al., “Electrochemiluminescence Lateral Flow Immunoassay Using Ruthenium(II) Complex‐Loaded Dendritic Mesoporous Silica Nanospheres for Highly Sensitive and Quantitative Detection of SARS‐CoV‐2 Nucleocapsid Protein,” Advanced Functional Materials 34 (2024): 2409632.

[13] M. Liu , W. Xu , Y. Tang , et al., “Tuning Atomically Dispersed Metal Sites in Nanozymes for Sensing Applications,” Angewandte Chemie, International Edition 64 (2025): 202424070. 10.1002/anie.202424070 39937141

[14] E. Kim , C.‐Y. Chen , M. J. Chu , M. F. Hamstra , W. E. Bentley , and G. F. Payne , “Proline‐Selective Electrochemiluminescence Detecting a Single Amino Acid Variation Between A1 and A2 β‐Casein Containing Milks,” Advanced Science 12 (2025): 2411956. 39644502 10.1002/advs.202411956 PMC11792022

[15] H. Gao , Y.‐L. Jia , J.‐B. Lin , et al., “Enhanced Aggregation‐Induced Delayed Electrochemiluminescence Triggered by Spatial Perturbation of Organic Dots,” Analytical Chemistry 96 (2024): 7780–7786. 38695093 10.1021/acs.analchem.4c01643

[16] Y.‐L. Jia , J.‐B. Lin , H. Gao , H.‐Y. Chen , and J.‐J. Xu , “Molecular Planar Rigidity Promoted Aggregation‐Induced Delayed Electrochemiluminescence of Organic Dots for Nucleic Acid Assay,” Analytical Chemistry 96 (2024): 18214–18220. 39485992 10.1021/acs.analchem.4c04413

[17] Y. Shen , X. Gao , H.‐J. Lu , C. Nie , and J. Wang , “Electrochemiluminescence‐Based Innovative Sensors for Monitoring the Residual Levels of Heavy Metal Ions in Environment‐Related Matrices,” Coordination Chemistry Reviews 476 (2023): 214927.

[18] Q.‐Q. Jiang , Y.‐J. Li , Q. Wu , et al., “Efficient Charge Transfer Driven Electrochemiluminescence in Heteroatom‐Involved Cocrystal Engineering for Detection of Uranyl Ions,” Analytical Chemistry 96 (2024): 19740–19749. 39574243 10.1021/acs.analchem.4c05011

[19] J. Dong , Y. Lu , Y. Xu , et al., “Direct Imaging of Single‐Molecule Electrochemical Reactions in Solution,” Nature 596 (2021): 244–249. 34381236 10.1038/s41586-021-03715-9

[20] D. Han , N. Sojic , and D. Jiang , “Spatial Profiling of Multiple Enzymatic Activities at Single Tissue Sections via Fenton‐Promoted Electrochemiluminescence,” Journal of the American Chemical Society 147 (2025): 9610–9619. 40063963 10.1021/jacs.4c17749

[21] S. Knežević , D. Han , B. Liu , D. Jiang , and N. Sojic , “Electrochemiluminescence Microscopy,” Angewandte Chemie, International Edition 63 (2024): 202407588. 10.1002/anie.202407588 38742673

[22] J. Descamps , C. Colin , G. Tessier , S. Arbault , and N. Sojic , “Ultrasensitive Imaging of Cells and Sub‐Cellular Entities by Electrochemiluminescence,” Angewandte Chemie, International Edition 62 (2023): 202218574. 10.1002/anie.202218574 36811514

[23] Y. Yan , P. Zhou , L. Ding , W. Hu , W. Chen , and B. Su , “T Cell Antigen Recognition and Discrimination by Electrochemiluminescence Imaging,” Angewandte Chemie, International Edition 62 (2023): 202314588. 10.1002/anie.202314588 37903724

[24] Z. Xing , X. Gou , L. P. Jiang , J. J. Zhu , and C. Ma , “An In Situ Investigation of the Protein Corona Formation Kinetics of Single Nanomedicine Carriers by Self‐Regulated Electrochemiluminescence Microscopy,” Angewandte Chemie, International Edition 62 (2023): 202308950. 10.1002/anie.202308950 37553293

[25] Z. Xing , X. Gou , R. Yang , et al., “Pre‐Label‐Free Three‐Dimensional Imaging of Cellular Bottom Topography with a Charge‐Lock Electrochemiluminescence Microscope,” Nano Letters 25 (2025): 7507–7516. 40289725 10.1021/acs.nanolett.5c01182

[26] W. Fu , M. Qi , Y. Rong , C. Lin , W. Guo , and B. Su , “Remote On‐Paper Electrochemiluminescence‐Based High‐Safety and Multilevel Information Encryption,” Angewandte Chemie, International Edition 64 (2025): 202420184. 10.1002/anie.202420184 39659206

[27] J. Chen , T. Wu , Y. Du , et al., “Iron‐Doped Cobalt Molybdate Enhanced Electrochemiluminescence Imaging for Dynamic Multilevel Information Encryption System Construction and Biomarker Sensing Analysis,” Angewandte Chemie, International Edition 64 (2025): 202507426. 10.1002/anie.202507426 40288990

[28] J.‐W. Oh , J.‐Y. Jeong , T.‐Y. Eom , S.‐D. Baek , and J.‐M. Myoung , “Enhanced Light‐Emission Efficiency of Multi‐Color Electrochemiluminescence Displays using Electrochemical Au Nanoparticle Catalysts with Three Dimensional ZnO Nanorod Electrodes,” Chemical Engineering Journal 416 (2021): 129202.

[29] S. Shin , Y. S. Park , S. Cho , et al., “Effect of Ion Migration in Electro‐Generated Chemiluminescence Depending on the Luminophore Types and Operating Conditions,” Chemical Science 9 (2018): 2480–2488. 29732124 10.1039/c7sc03996d PMC5909676

[30] S. Lee , W. S. Cho , J. Y. Park , et al., “Water Washable and Flexible Light‐Emitting Fibers Based on Electrochemiluminescent Gels,” ACS Applied Materials & Interfaces 14 (2022): 17709–17718. 35389205 10.1021/acsami.2c01438

[31] X. Li , J. F. Lovell , J. Yoon , and X. Chen , “Clinical Development and Potential of Photothermal and Photodynamic Therapies for Cancer,” Nature Reviews Clinical Oncology 17 (2020): 657–674. 10.1038/s41571-020-0410-2 32699309

[32] S. Liu , H. Yuan , H. Bai , et al., “Electrochemiluminescence for Electric‐Driven Antibacterial Therapeutics,” Journal of the American Chemical Society 140 (2018): 2284–2291. 29353473 10.1021/jacs.7b12140

[33] M.‐M. Chen , C.‐H. Xu , W. Zhao , H.‐Y. Chen , and J.‐J. Xu , “Single Cell Imaging of Electrochemiluminescence‐Driven Photodynamic Therapy,” Angewandte Chemie, International Edition 61 (2022): 202117401. 10.1002/anie.202117401 35165987

[34] Y. Yuan , D. Yan , and R. Duan , “A Flexible Antibacterial Gel Electrochemiluminescence Device for Monitoring and Therapy of Chronic Diabetic Wounds,” Aggregate 6 (2024), 703.

[35] K. G. Cho , J. I. Lee , S. Lee , K. Hong , M. S. Kang , and K. H. Lee , “Light‐Emitting Devices Based on Electrochemiluminescence Gels,” Advanced Functional Materials 30 (2020): 1907936.

[36] H. C. Moon , T. P. Lodge , and C. D. Frisbie , “Solution‐Processable Electrochemiluminescent Ion Gels for Flexible, Low‐Voltage, Emissive Displays on Plastic,” Journal of the American Chemical Society 136 (2014): 3705–3712. 24517258 10.1021/ja5002899

[37] J. I. Lee , H. Choi , S. H. Kong , et al., “Visco‐Poroelastic Electrochemiluminescence Skin with Piezo‐Ionic Effect,” Advanced Materials 33 (2021): 2100321. 10.1002/adma.202100321 34060148

[38] D.‐K. Kwon and J.‐M. Myoung , “Ion Gel‐Based Flexible Electrochemiluminescence Full‐Color Display with Improved Sky‐Blue Emission using a Mixed‐Metal Chelate System,” Chemical Engineering Journal 379 (2020): 122347.

[39] J. H. Kwon , Y. M. Kim , and H. C. Moon , “Porous Ion Gel: A Versatile Ionotronic Sensory Platform for High‐Performance, Wearable Ionoskins with Electrical and Optical Dual Output,” ACS Nano 15 (2021): 15132–15141. 34427425 10.1021/acsnano.1c05570

[40] D.‐K. Kwon and J.‐M. Myoung , “Wearable and Semitransparent Pressure‐Sensitive Light‐Emitting Sensor Based on Electrochemiluminescence,” ACS Nano 14 (2020): 8716–8723. 32644780 10.1021/acsnano.0c03186

[41] O. E. Geiculescu , B. B. Hallac , R. V. Rajagopal , et al., “The Effect of Low‐Molecular‐Weight Poly(ethylene glycol) (PEG) Plasticizers on the Transport Properties of Lithium Fluorosulfonimide Ionic Melt Electrolytes,” The Journal of Physical Chemistry B 118 (2014): 5135–5143. 24773589 10.1021/jp500826c

[42] Y. Li , X. Li , S. Zhang , et al., “Autonomic Self‐Healing of PEDOT:PSS Achieved Via Polyethylene Glycol Addition,” Advanced Functional Materials 30 (2020): 2002853.

[43] Y. Li , S. Zhang , X. Li , V. R. N. Unnava , and F. Cicoira , “Highly Stretchable PEDOT:PSS Organic Electrochemical Transistors Achieved via Polyethylene Glycol Addition,” Flexible and Printed Electronics 4 (2019): 044004.

[44] Q. Lu , T. Fang , C. Ye , et al., “Highly Conductive Liquid Metal Emulsion Gels for Three‐Dimensionally Printed Stretchable Electronics,” Advanced Science 12 (2025): 03449. 10.1002/advs.202503449 PMC12462967 40619609

[45] H. Yee , J. I. Lee , D. M. Park , et al., “Extending the Operational Lifetime of Electrochemiluminescence Devices by Installing a Floating Bipolar Electrode,” Small 20 (2024): 2307190. 10.1002/smll.202307190 38009522

[46] C. C. P. Cid , E. R. Spada , and M. L. Sartorelli , “Effect of the Cathodic Polarization on Structural and Morphological Proprieties of FTO and ITO Thin Films,” Applied Surface Science 273 (2013): 603–606.

[47] L. Liu , S. Yellinek , I. Valdinger , A. Donval , and D. Mandler , “Important Implications of the Electrochemical Reduction of ITO,” Electrochimica Acta 176 (2015): 1374–1381.

[48] S. Macher , M. Rumpel , M. Schott , U. Posset , G. A. Giffin , and P. Löbmann , “Avoiding Voltage‐Induced Degradation in PET‐ITO‐Based Flexible Electrochromic Devices,” ACS Applied Materials & Interfaces 12 (2020): 36695–36705. 32664716 10.1021/acsami.0c07860

[49] H. Oh , Y. M. Kim , U. Jeong , and H. C. Moon , “Balancing the Concentrations of Redox Species to Improve Electrochemiluminescence by Tailoring the Symmetry of the AC Voltage,” ChemElectroChem 5 (2018): 2836–2841.

[50] J. Zhang , X. Mao , Q. Jia , et al., “Body‐Worn and Self‐Powered Flexible Optoelectronic Device for Metronomic Photodynamic Therapy,” npj Flexible Electronics 8 (2024): 60.

[51] M. Li , S. Zhou , Q. Yu , et al., “A Sprayable TQ/Ce6@SAB/F‐Gel for Accelerating Wound Healing via Hypoxia‐Tolerant Photodynamic Therapy and Immune–Metabolic Pathway,” Biomaterials 325 (2026): 123602. 40782448 10.1016/j.biomaterials.2025.123602

[52] Z.‐C. Hu , B. Wang , X.‐G. Zhou , et al., “Golgi Apparatus‐Targeted Photodynamic Therapy for Enhancing Tumor Immunogenicity by Eliciting NLRP3 Protein‐Dependent Pyroptosis,” ACS Nano 17 (2023): 21153–21169. 37921421 10.1021/acsnano.3c05005

[53] E.‐S. Ko , J. I. Lee , H. C. Lim , et al., “Pulsed Driving Methods for Enhancing the Stability of Electrochemiluminescence Devices,” ACS Photonics 5 (2018): 3723–3730.

[54] J. I. Lee , D. Kang , S. H. Kong , et al., “Dynamic Interplay between Transport and Reaction Kinetics of Luminophores on the Operation of AC‐Driven Electrochemiluminescence Devices,” ACS Applied Materials and Interfaces 10 (2018): 41562–41569. 30398048 10.1021/acsami.8b13680

[55] A. Hak , M. S. Ali , S. A. Sankaranarayanan , V. R. Shinde , and A. K. Rengan , “Chlorin e6: A Promising Photosensitizer in Photo‐Based Cancer Nanomedicine,” ACS Applied Bio Materials 6 (2023): 349–364. 10.1021/acsabm.2c00891 36700563

[56] X. Chen , B. B. Mendes , Y. Zhuang , et al., “A Fluorinated BODIPY‐Based Zirconium Metal–Organic Framework for In Vivo Enhanced Photodynamic Therapy,” Journal of the American Chemical Society 146 (2024): 1644–1656. 38174960 10.1021/jacs.3c12416 PMC10797627

