# Supplementary material for: Electrochemiluminescence Drives Photodynamic Therapy In Vivo
Source: Adv Sci (Weinh). 2025 Dec 22;13(14):e12027. doi: 10.1002/advs.202512027 (PMC12970228; doi:10.1002/advs.202512027)
Supplement: Supplementary file 1 — Supporting File 1: advs73531‐sup‐0001‐SuppMat.pdf. [file ADVS-13-e12027-s002.docx]

Supporting Information

Electrochemiluminescence Drives Photodynamic Therapy *In Vivo*

Jia-Bao Lin,^1#^ Ling-Ling Xu,^2#^ Yong Liu,^1^ Hang Gao,^2*^ Hong-Yuan Chen,^1^ Jing-Juan Xu^1*^

*^1^*State Key Laboratory of Analytical Chemistry for Life Science, School of Chemistry and Chemical Engineering, Nanjing University, Nanjing 210023, China

*^2^*School of Chemistry and Chemical Engineering, Yangzhou University, Yangzhou 225002, China

^#^These authors contributed equally to this work.

*Corresponding Authors: [xujj@nju.edu.cn](mailto:xujj@nju.edu.cn) (Jing-Juan Xu); [gaohang@yzu.edu.cn](mailto:gaohang@yzu.edu.cn) (Hang Gao).

**
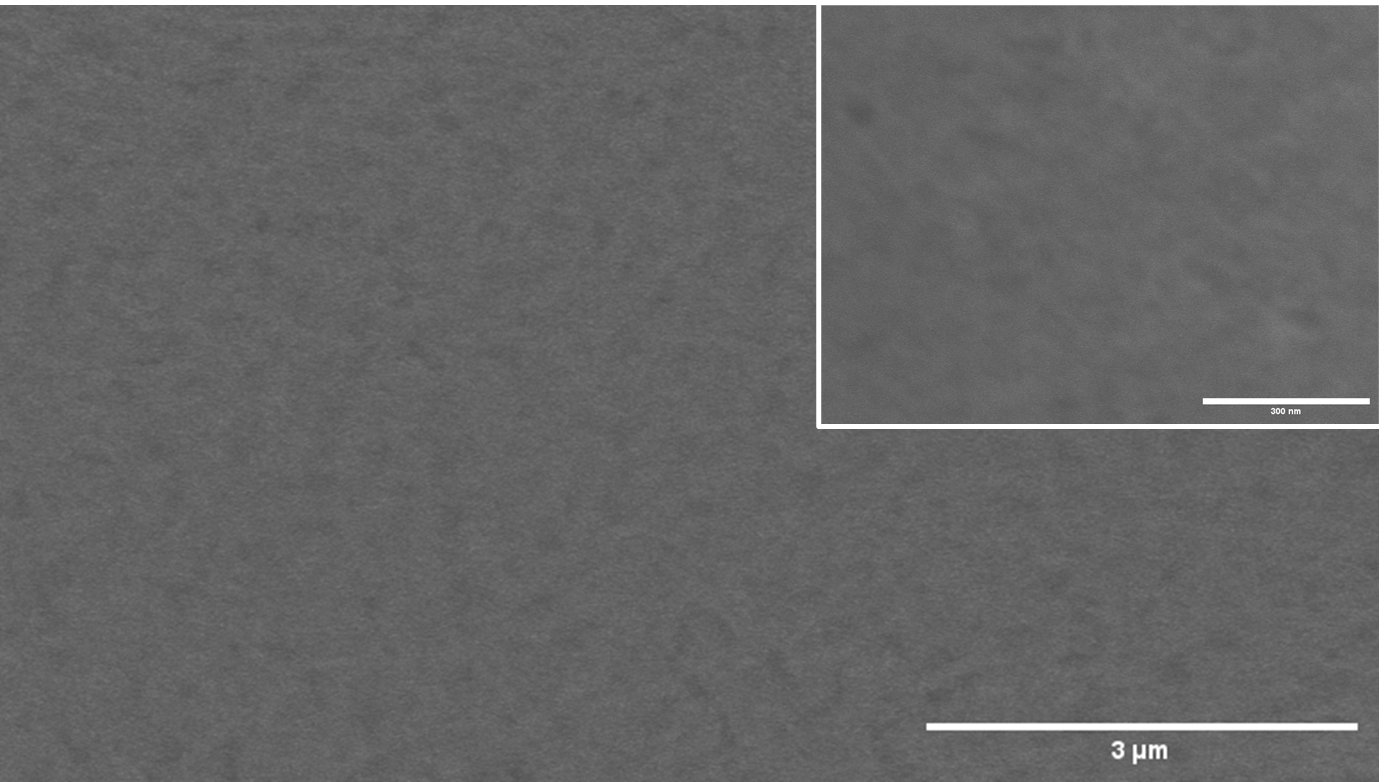
**

**Figure S1.** SEM images of the ECL gel prepared by heat treatment.

**Figure S2**. Viscosity versus shear rate of the ECL gel.

**
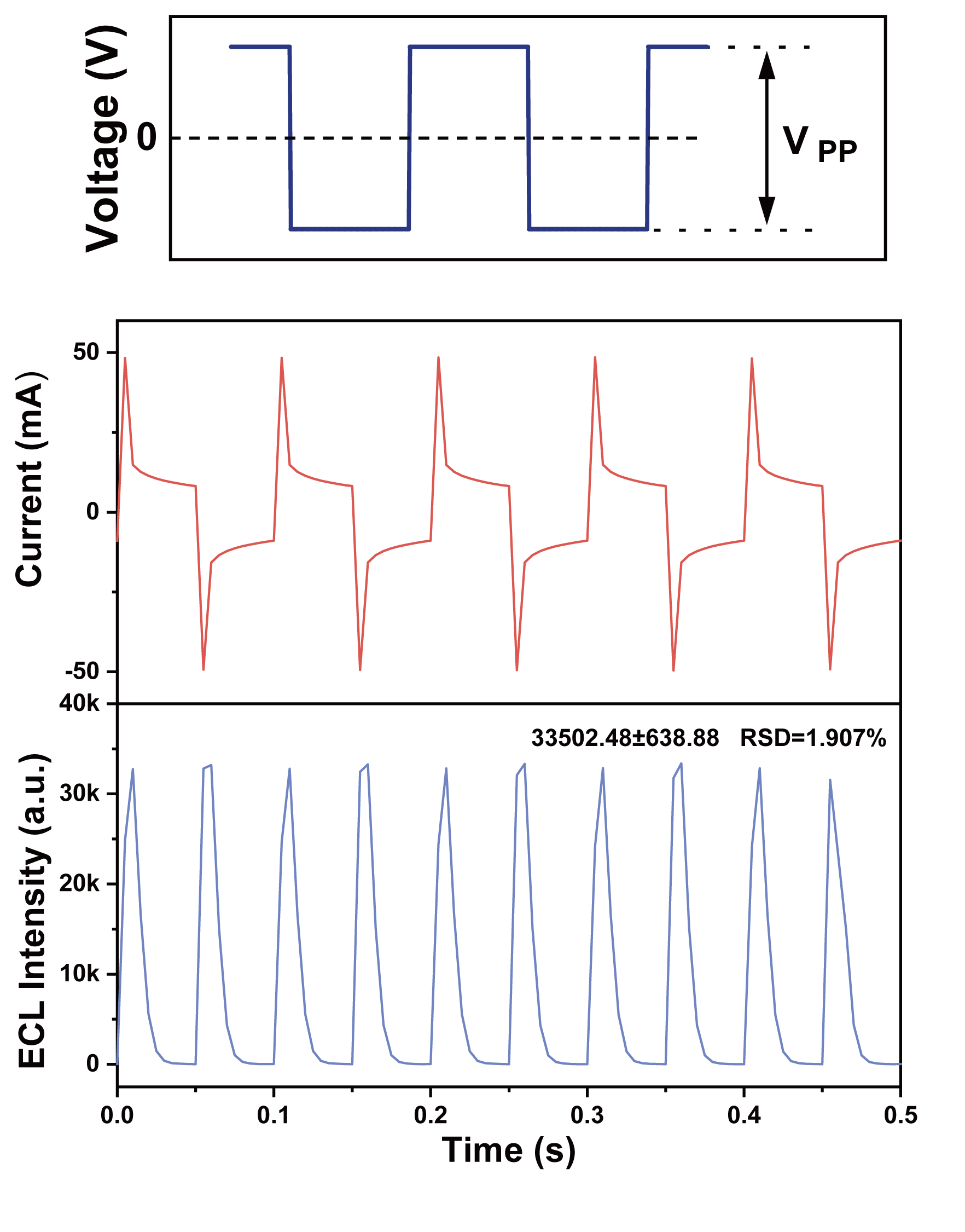
**

**Figure S3.** Current and ECL (PMT = 150 V) curves for the ECLD under a square wave voltage signal (V_PP_ = 5.6 V) at a frequency of 10 Hz.

When an alternating current (AC) voltage is applied to ECLD, the Ru(bpy)_3_^2+^ within the ECL gel undergoes oxidation or reduction reactions at the electrode surface, as follows:

$Ru(bpy)_{3}^{2+}-e^{-}\to Ru(bpy)_{3}^{3+} (positive)$ (1)

$Ru(bpy)_{3}^{2+}+e^{-}\to Ru(bpy)_{3}^{+} (negative)$ (2)

As shown in Scheme 1, when the applied voltage is switched, opposite redox reactions occur at each electrode. For example, when the voltage switches from positive to negative, Ru(bpy)_3_^3+^ undergoes the following reduction reactions at the electrode interface:

$Ru(bpy)_{3}^{3+}+e^{-}\to Ru(bpy)_{3}^{2+} (negative)$ (3)

$Ru(bpy)_{3}^{2+}+e^{-}\to Ru(bpy)_{3}^{+} (negative)$ (2)

Due to the concentration gradient, the generated Ru(bpy)_3_^+^ and the previously formed Ru(bpy)_3_^3+^ diffuse toward each other, and then undergo an electron transfer reaction:

$Ru(bpy)_{3}^{+}+Ru(bpy)_{3}^{3+}\to Ru(bpy)_{3}^{2+ *}+Ru(bpy)_{3}^{2+}$ (4)

Ultimately, the excited Ru(bpy)_3_^2+*^ is generated at both electrodes and emits light through the radiative transition:

$Ru(bpy)_{3}^{2+ *}\to Ru(bpy)_{3}^{2+}+hv$ (5)

With the further switch of AC voltage, Ru(bpy)₃²⁺-regenerated through radiative deactivation of Ru(bpy)_3_^2+*^-is sequentially oxidized at the anode to Ru(bpy)₃³⁺ and reduced at the cathode to Ru(bpy)₃⁺, as described by eqs. 1 and 2, respectively.

Next, the newly generated redox species and the previous redox species undergo a diffusion and an electron transfer analogous to that in eq. 4. Excited species Ru(bpy)_3_^2+*^ are thus generated, subsequently emitting photons, as delineated in eq. 5.

**Figure S4**. CV and ECL curves of Ru(bpy)_3_Cl_2_ (0.1 mM) in 0.1 M phosphate buffer saline (PBS; 7.4 pH) containing 25 mM tri-*n*-propylamine (TPrA) as co-reactant.


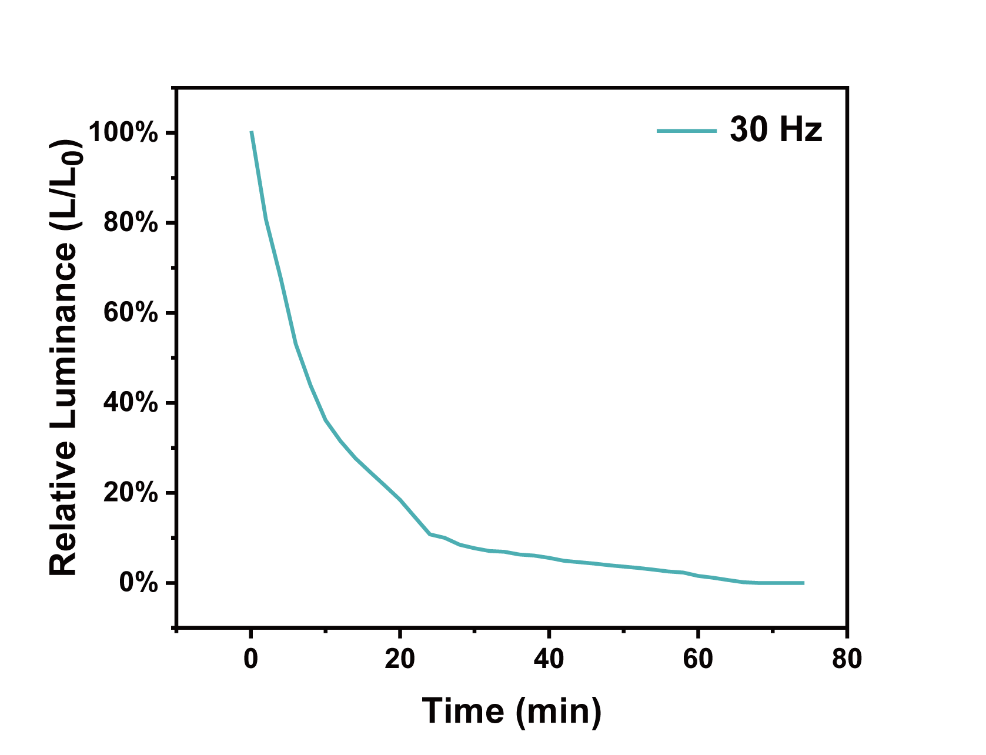


**Figure S5.** Relative ECL luminance (L/L_0_, where L_0_ represents the initial luminance) of the ECLD as a function of continuous operating time at V_PP_ = 5.6 V and a frequency of 30 Hz.


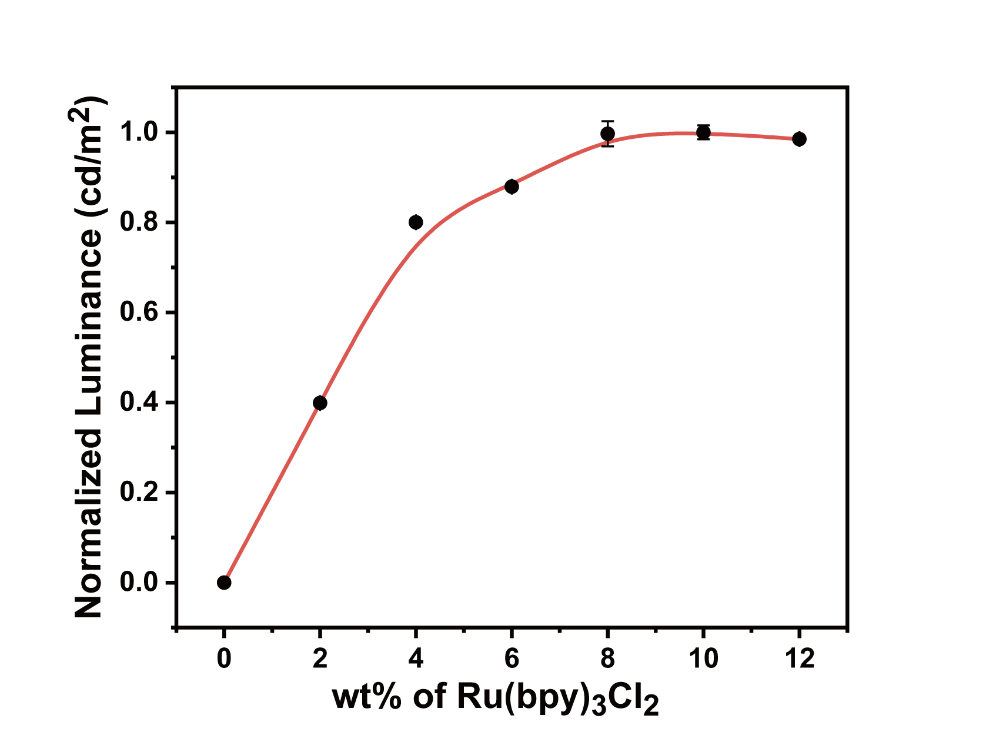


**Figure S6.** Normalized ECL luminance of the ECLD as a function of Ru(bpy)_3_Cl_2_ concentration at V_PP_ = 5.6 V and a frequency of 60 Hz (*n* = 3; mean ± SD).


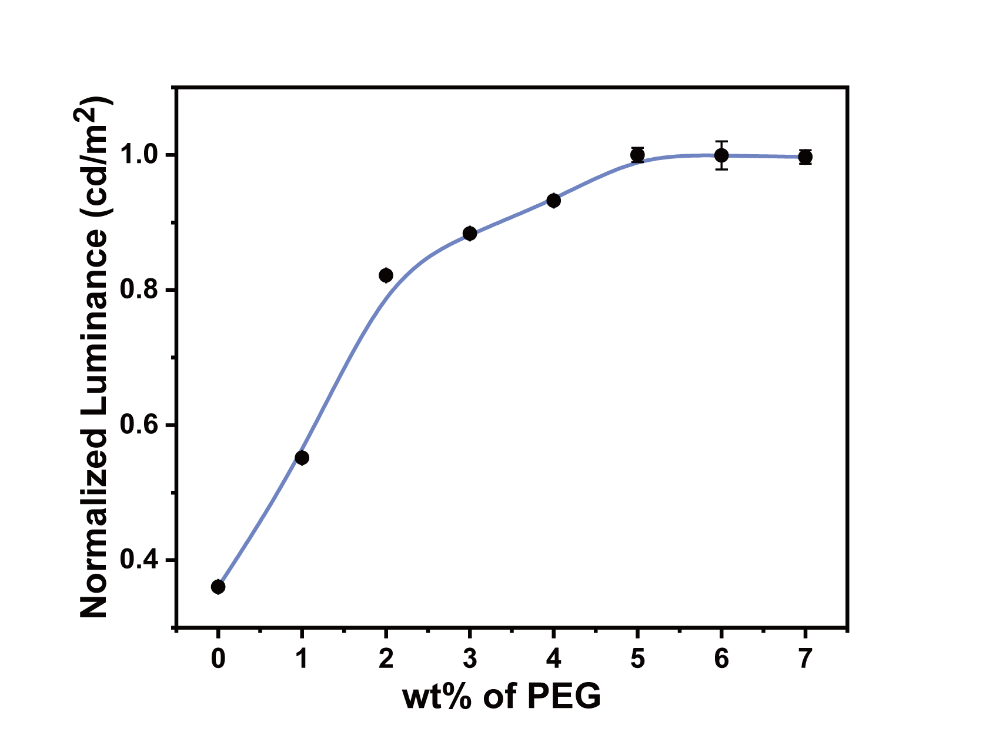


**Figure S7.** Normalized ECL luminance of the ECLD containing 8 wt% Ru(bpy)_3_Cl_2_ as a function of PEG concentration at V_PP_ = 5.6 V and a frequency of 60 Hz (*n* = 3; mean ± SD).

**Figure S8**. Relative luminance (L/L_0_, where L_0_ represents the initial luminance) of the ECLD without PEG during continuous operation for 30 min.

**
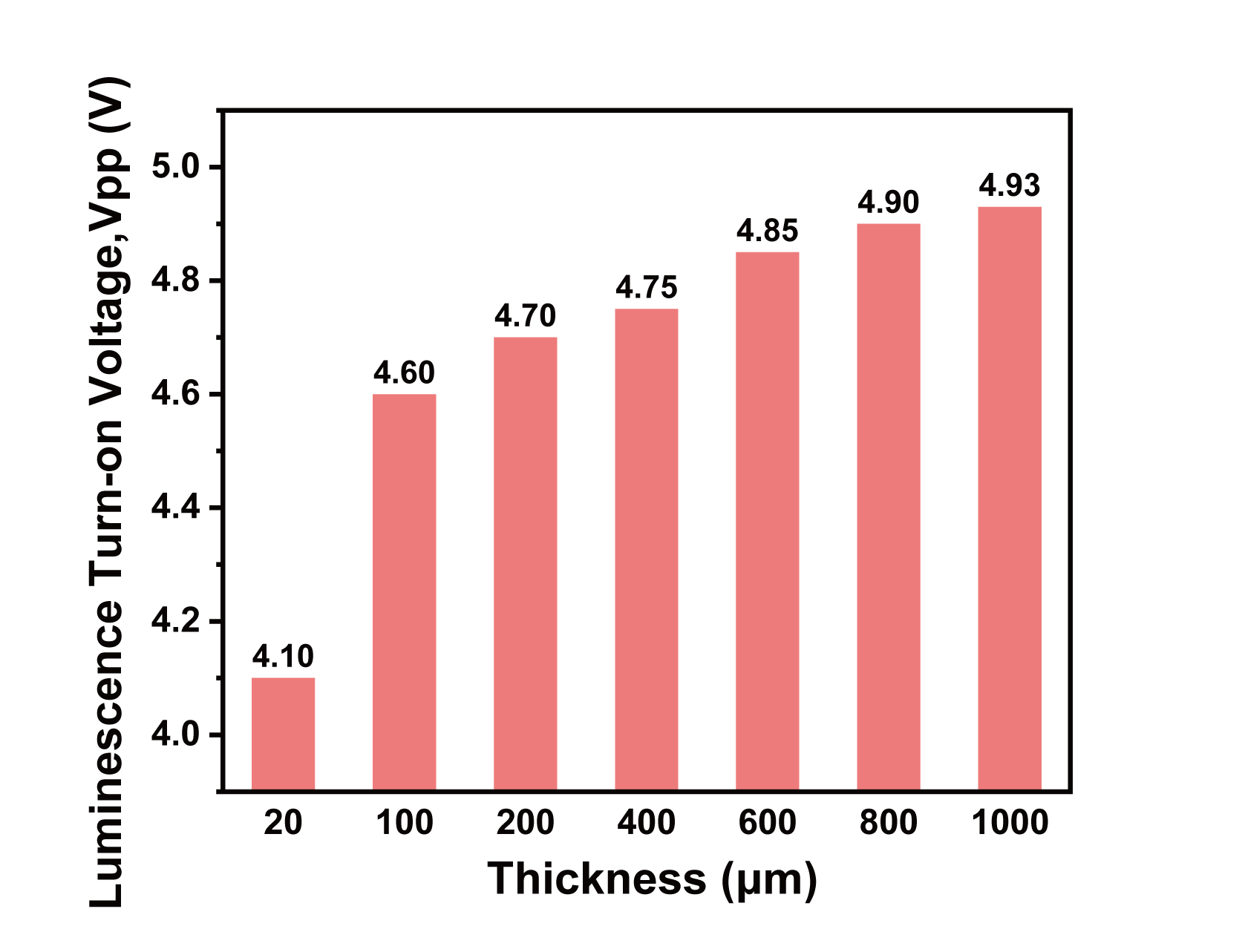
**

**Figure S9.** Luminescence turn-on voltage (V_PP_) of the ECLD as a function of thickness of ECL gel at 60 Hz.

**
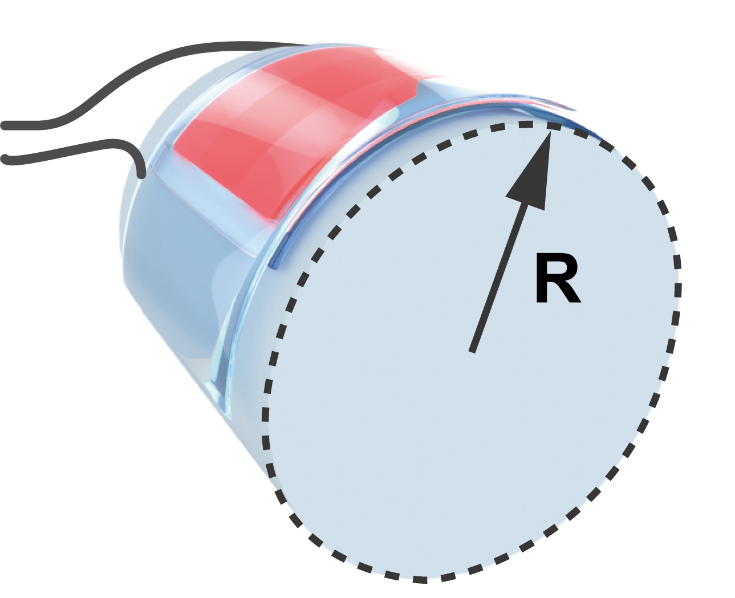
**

**Figure S10.** Schematic illustration of the curvature radius (R) of the flexible ECLD during bending. The ECLD was attached to cylindrical objects of different diameters, and its luminance was measured at V_PP_ = 5.6 V and a frequency of 60 Hz.

**
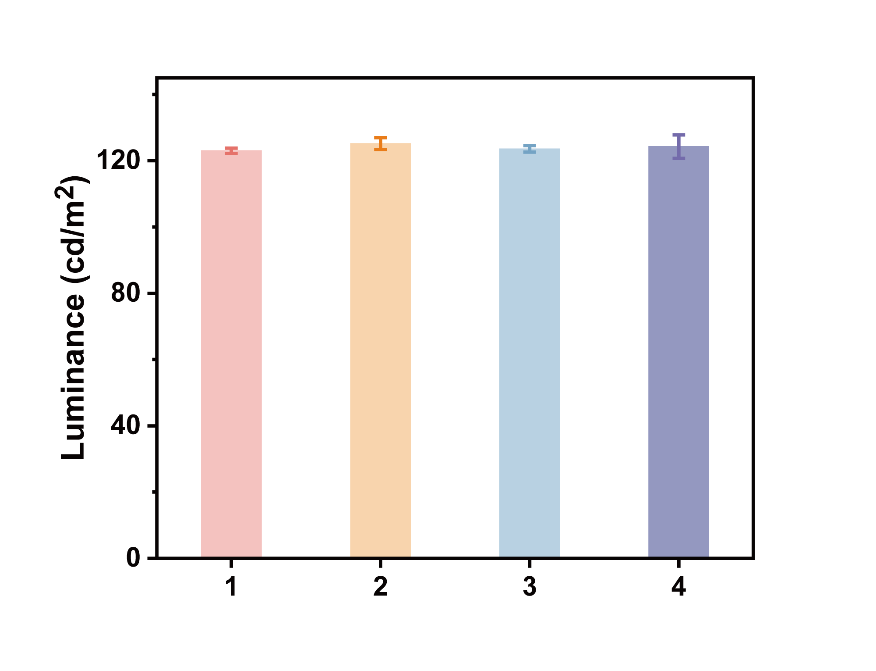
**

**Figure S11.** Stability of the ECLD: ECL luminance (n = 5; mean ± SD) of four fabricated ECLDs with identical ECL gel areas (60 Hz, V_PP_=5.6 V).


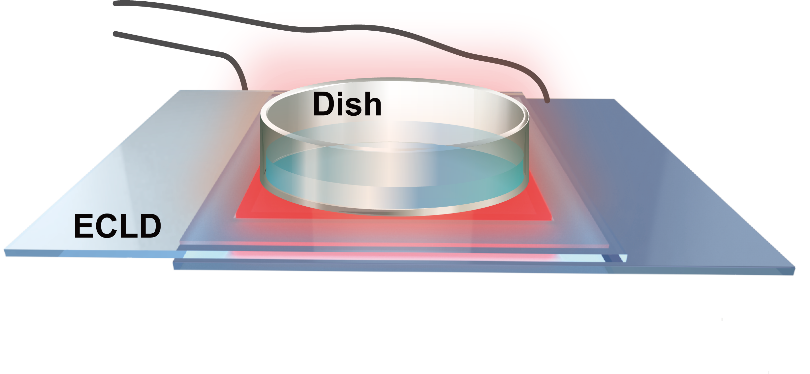


**Figure S12.** Schematic illustration of the experimental design for evaluating ROS generation in ECL-PDT. The solution containing DPBF and Ce6 was added in a dish, followed by ECLD irradiation (60 Hz, V_PP_=5.6 V) through the bottom of the dish.


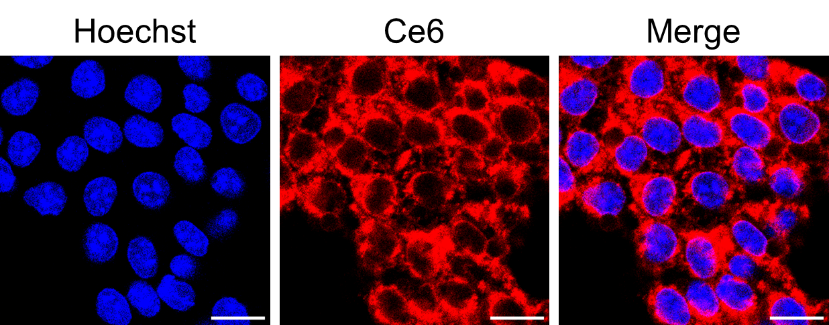


**Figure S13.** CLSM images of intracellular Ce6 fluorescence in 4T1 cells following an 8-hour incubation with Ce6 (100 μM), indicating the cellular uptake of Ce6. The scale bar is 20 μm.


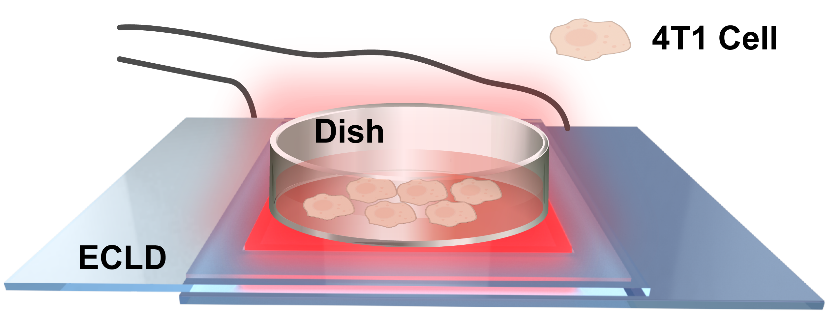


**Figure S14.** Schematic illustration of the experimental design for evaluating intracellular ROS generation in 4T1 cells.


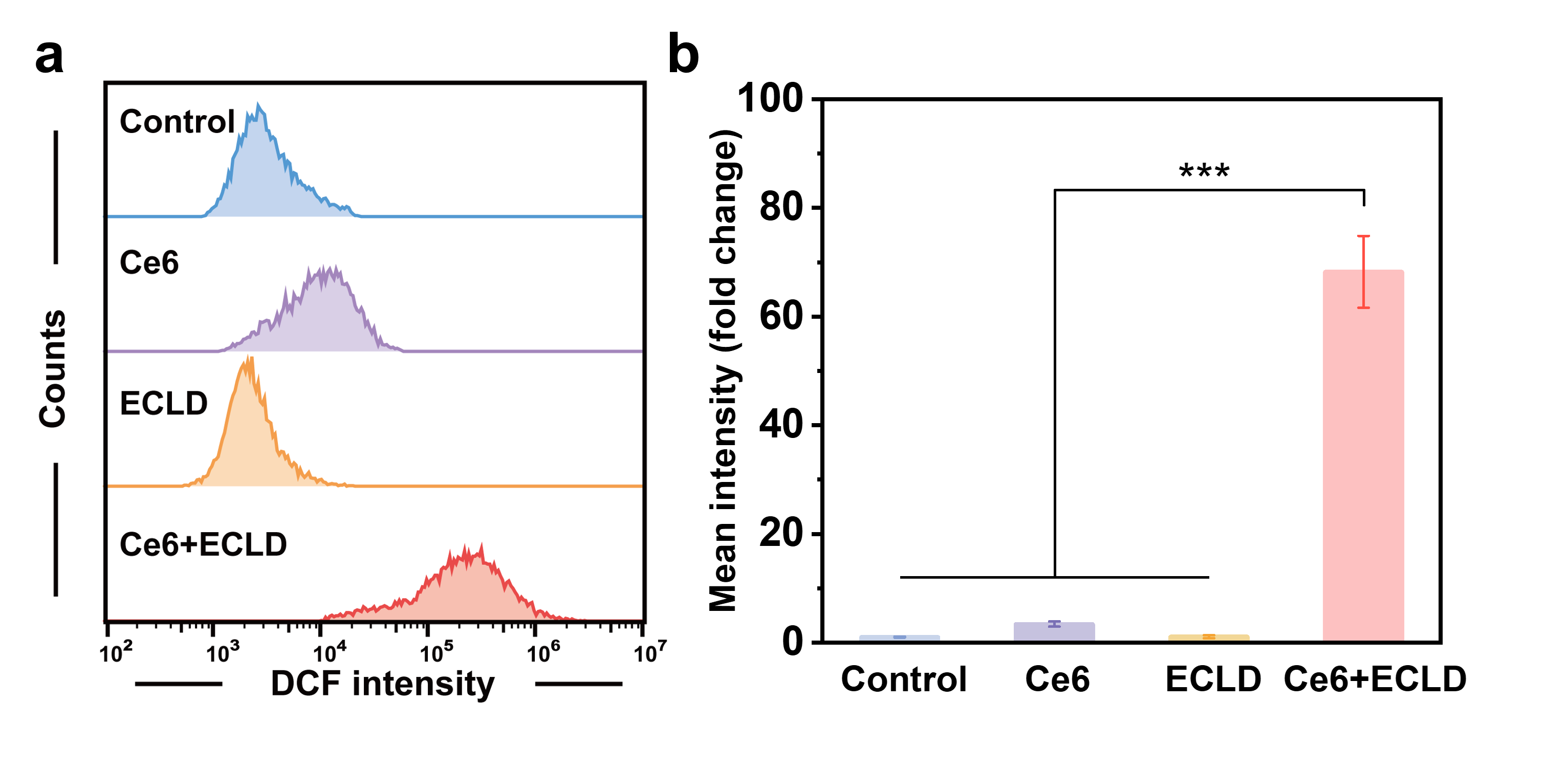


**Figure S15**. (a) Histograms of DCF intensity for 4T1 cells measured by flow cytometry after different treatments. (b) Normalized DCF fluorescent intensity (*n* = 3; mean ± SD), statistical analyses were carried out using one-way analysis of variance (ANOVA) with Tukey's test. ****P* < 0.001.

**
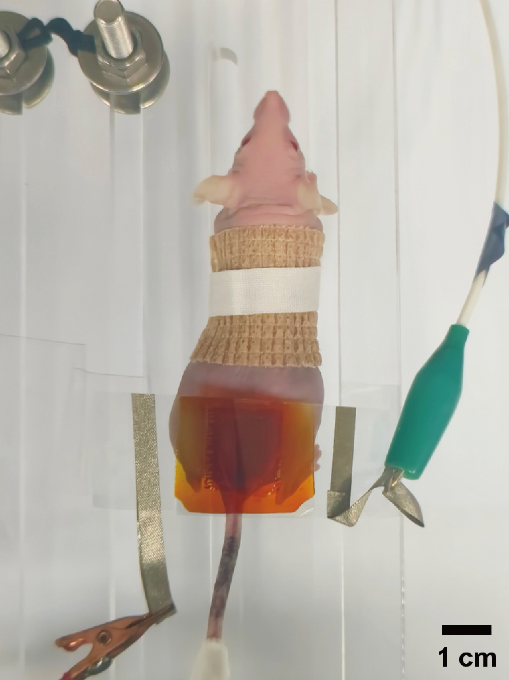
**

**Figure S16.** The photo of tumor-bearing mouse under ECL-PDT treatment.


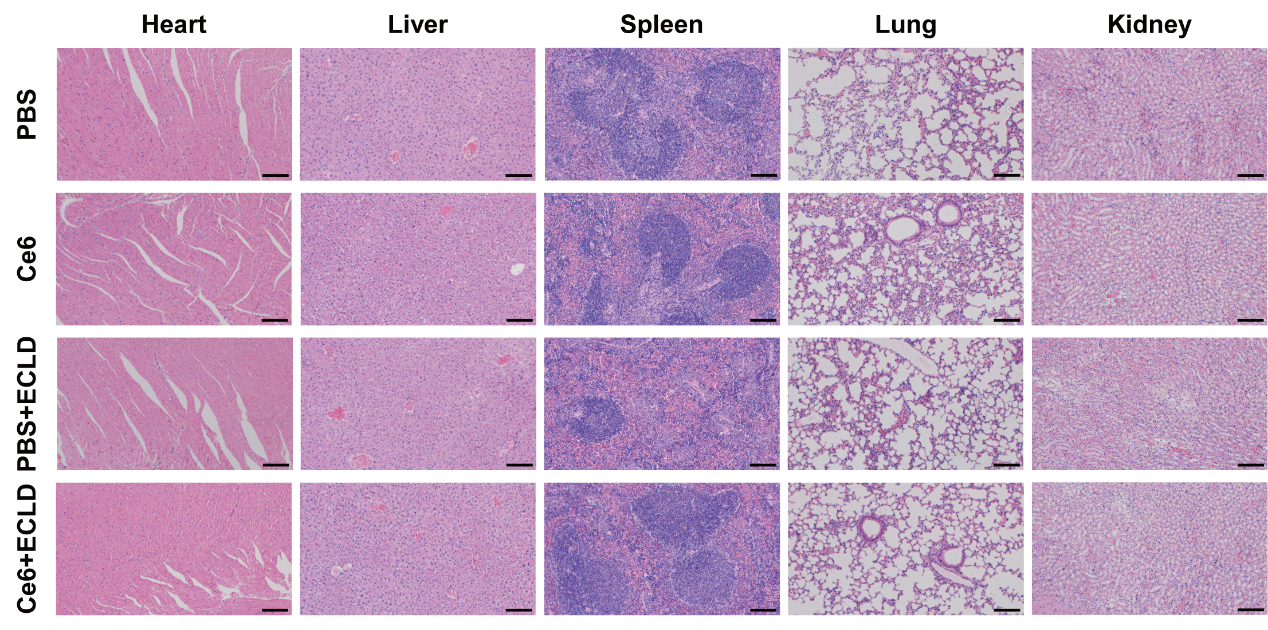


**Figure S17.** H&E staining images of major organs of mice after different treatments. The scale bar is 100 μm.


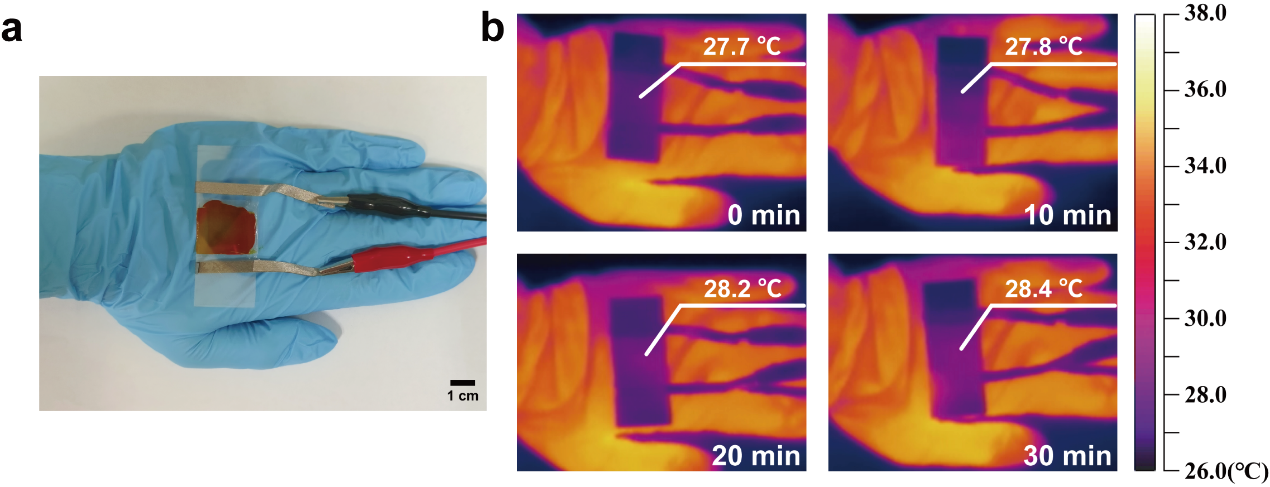


**Figure S18**. (a) Photograph of the ECLD prior to thermal imaging acquisition. (b) Infrared (IR) thermal images of the ECLD treated with AC voltage at a frequency of 60 Hz and V_PP_ = 5.6 V.
